# A cationic single-chain bolalipid forms stable vesicles with distinct interfacial behavior

**DOI:** 10.1016/j.bpj.2026.03.044

**Published:** 2026-03-26

**Authors:** Preeti Gahtori, Hossein Varghaei, Bibhas Hazra, Christian Jankovic, Jonathan Strobl, Harpreet Kaur, Sara Khamis, Frederick G. West, Julianne M. Gibbs, Sheref S. Mansy

**Affiliations:** 1 Department of Chemistry, University of Alberta, Edmonton, AB, Canada

## Abstract

The ability of single-chain bolalipids (or bolaamphiphiles) to self-assemble into vesicular structures remains poorly characterized. Here, we report the synthesis and self-assembly behavior of a new class of proline-based single-chain lipids, a symmetric bipolar molecule (2Pro-C18:1) and, for comparison, a unipolar analog (1Pro-C18:1), both bearing an unsaturated C18 hydrocarbon chain. Confocal fluorescence microscopy, encapsulation assays, and fluorescence spectroscopy demonstrate that both amphiphiles form stable vesicles capable of entrapping small molecules. Vesicle formation occurs over a broad range of pH values, with the bolalipid favoring higher pH, consistent with differences in apparent pK_a_ determined from zeta potential measurements. At the air-water interface, 2Pro-C18:1 exhibits lower surface activity than 1Pro-C18:1, and vibrational sum frequency generation spectroscopy is consistent with a bent or U-shaped interfacial conformation for the bolalipid, in contrast to a more extended geometry for the unipolar analog. Both lipids display dynamic exchange between interfacial and bulk-associated aggregates at higher concentrations, with 2Pro-C18:1 exhibiting more complex concentration-dependent behavior. Taken together, these results demonstrate that single-chain bolalipids can form stable, dynamic aggregates with bulk and interfacial properties that are governed primarily by hydrophobic chain effects modulated by headgroup architecture. These findings provide insight into molecular design principles for functional amphiphilic systems.

## Graphical abstract

**Figure:**
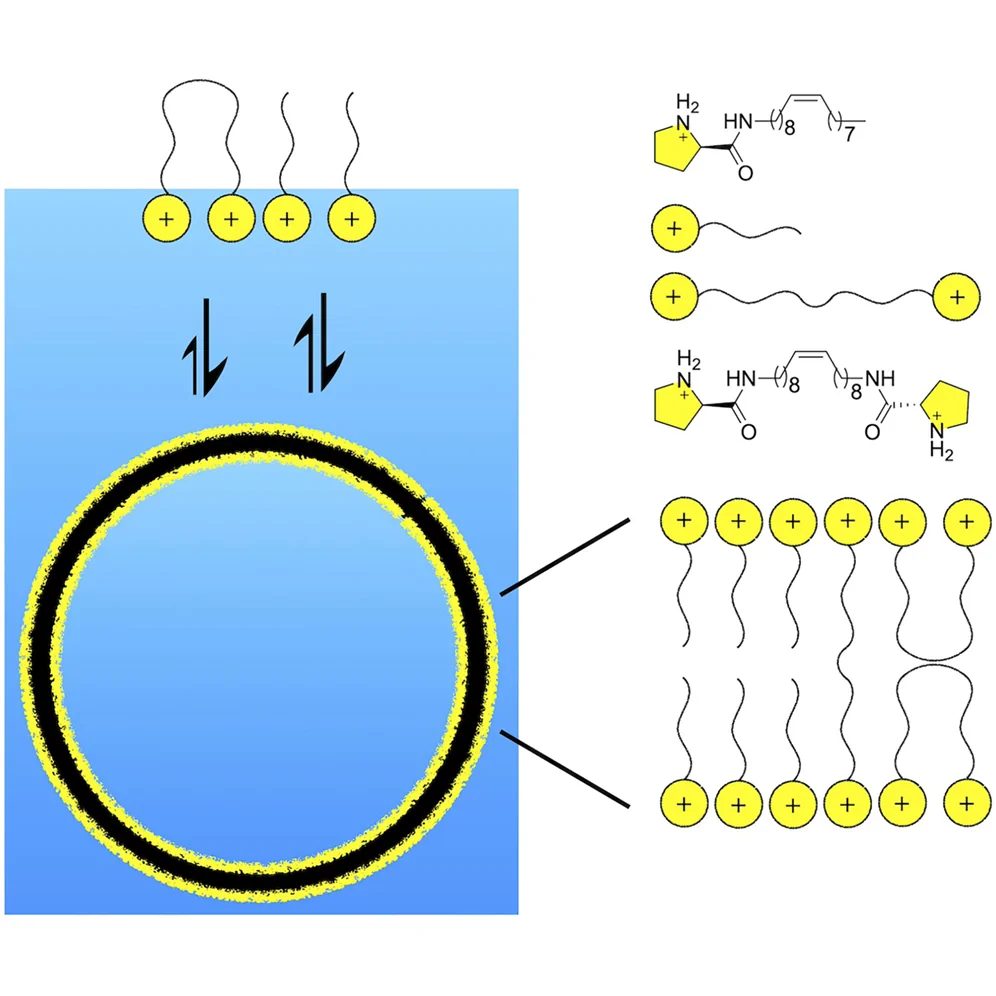

## Significance

Biological membranes are typically built from lipids bearing one polar headgroup and two hydrophobic tails, but synthetic chemistry allows for alternative architectures. Here, we show that single-chain bolalipids with proline headgroups self-assemble into stable vesicles while exhibiting distinct interfacial behavior relative to a unipolar analog. By combining fluorescence microscopy, surface tension measurements, and vibrational sum frequency generation spectroscopy, we demonstrate that molecular symmetry and headgroup chemistry modulate interfacial organization and concentration-dependent exchange between bulk aggregates and the air-water interface. These findings expand current understanding of lipid self-assembly and provide design principles for dynamic membrane systems relevant to artificial cells and soft materials.

## Introduction

Biological membrane lipids typically consist of a single hydrophilic headgroup and two hydrophobic chains. Such lipids have been used for the synthesis of lipid nanoparticles (1) and vesicles (2), as components of artificial cells (3,4) and model protocells (5,6,7), and as a medium for the reconstitution of proteins (8). However, exceptions to this typical unipolar structure exist. For example, lipids with two hydrophobic chains and two hydrophilic headgroups (bipolar lipids or bolalipids) are found in Archaea (9). These bolalipids form monolayer rather than bilayer membranes, with hydrophobic chains spanning the entire width of the membrane and hydrophilic headgroups exposed to water on opposite sides, a structure thought to provide exceptional stability under extreme conditions such as high acidity, salinity, temperature, and pressure (9,10,11).

The ability of lipids to self-assemble into stable vesicles strongly depends on their molecular geometry (12). Conventional two-chain unipolar biological lipids form stable vesicles because their cylindrical shape and the large surface area provided by the two hydrophobic chains keeps the lipids firmly embedded in the membrane (13). Single-chain lipids can also form membranes (14), although the resulting vesicles are typically more dynamic and less stable than those formed by conventional two-chain lipids (15). Bolalipids with two hydrophobic chains frequently assemble into more rigid and stable aggregates, such as vesicles (16), nanofibers (17), nanotubes (18), and ribbon-like aggregates (19), in a manner dependent on their chain length, saturation, and environmental conditions. Bolalipids with two polar headgroups and one hydrophobic chain, unlike archaeal lipids with two hydrophobic chains, generally assemble into micelles or fibers that contain hydrophobic interiors rather than the aqueous interiors of bilayer structures (20,21). However, the vesicle-forming behavior of this single-chain category of bolalipids remains largely unexplored. Single-chain bolalipids are particularly intriguing because such lipids could potentially form vesicles that combine the mechanical stability typically associated with bolalipids with the enhanced membrane dynamics and flexibility of single-chain lipids, offering opportunities to design vesicles with tunable properties.

In this study, we synthesized a series of proline-based single-chain bolalipids with alkyl chain lengths ranging from C8 to C18, in which the hydrophilic proline headgroup is linked to the alkyl chain via the carboxylate of the proline. As a cyclic amino acid, proline introduces conformational constraints that can influence self-assembly behavior (22). Among the lipids examined, confocal fluorescence microscopy revealed that only the unsaturated C18:1 bolalipid (2Pro-C18:1) formed stable vesicular assemblies, with vesicle formation strongly modulated by the pH of the solution. Based on this result, we focused on comparing the bulk self-assembly and interfacial behavior of 2Pro-C18:1 with the structural analog 1Pro-C18:1, a unipolar lipid that contained a single Pro headgroup. Both lipids formed stable vesicles with similar critical aggregation concentrations (CACs) and were capable of encapsulating fluorophore. However, their interfacial behavior differed with surface tension measurements indicating more complex concentration-dependent behavior for 2Pro-C18:1. To probe interfacial organization at the molecular level, we employed vibrational sum frequency generation (vSFG) spectroscopy, a second-order nonlinear optical technique that selectively interrogates vibrational modes at interfaces (23,24). Together, these measurements indicate dynamic exchange between interfacial and bulk-associated lipid species and provide mechanistic insight into the self-assembly of single-chain bolalipids in both bulk solution and at interfaces.

## Materials and methods

### Chemicals

All chemicals were purchased from Sigma-Aldrich and used without further purification. All reactions were carried out in flame-dried glassware under a nitrogen atmosphere using solvents dried by a solvent purification system. Thin-layer chromatography analyses were conducted on silica gel 60 F254 aluminum plates (Millipore-Sigma), and column chromatography was performed using 230–400 mesh silica gel. ^1^H NMR spectra were recorded at 400 or 500 MHz, and coupling constants (*J*) are reported in Hertz (Hz). ^13^C NMR spectra were recorded at 100 or 125 MHz. Chemical shifts are referenced to the residual nondeuterated solvent signal of CDCl_3_ (s, 7.26 ppm, ^1^H; t, 77.06 ppm, ^13^C) as the internal standard and are reported on the δ scale (ppm). Multiplicities on ^1^H NMR are reported using standard notation: (br), apparent (ap), multiplet (m), singlet (s), doublet (d), triplet (t), quartet (q), pentet (p), etc. HRMS data were recorded using the Agilent Technologies 6220 oaTOF (ESI) or the Kratos MS50G (EI).

### Confocal fluorescence microscopy

Thin lipid films were individually prepared by rotary evaporation in a round-bottom flask of either 1Pro-C18:1 or 2Pro-C18:1 in 1:1 CHCl_3_:CH_3_OH. The lipid films were kept at 4°C overnight. The next day the lipid films were hydrated with deionized water (Milli-Q H_2_O, Synergy UV water purification system, Merck) to 40 mM lipid. The solution was then diluted 2-fold with a buffered solution to give a final concentration of 0.2 M bicine or Bis-tris propane supplemented with 50 mM NaCl and 0.25 mM HPTS (8-hydroxypyrene-1,3,6-trisulfonic acid) and tumbled for 30 min. The samples were then diluted again 2-fold before imaging. The final concentration of lipid was 10 mM. The Olympus FluoView FV3000 Confocal Laser Scanning Microscope (Olympus, USA) at the Advanced Microscopy Facility, Department of Biological Sciences, was used for imaging. Images were acquired using 488-nm laser diode excitation and analyzed with FV31S-SW Viewer software.

### Fluorescence spectroscopy

Nile Red was added from a 1 mM stock in acetone to a final concentration of 5 μM to a solution containing 10 mM 1Pro-C18:1 or 2Pro-C18:1 in 0.2 M bicine, 50 mM NaCl, pH 8.0. Fluorescence spectra with excitation at 530 nm were collected using a SpectraMax i3x (Molecular Devices) in the Microbiology Lab, Chemistry Department facility. Emission maxima of 600–605 nm, 625–630 nm, and 635–640 nm are consistent with the presence of oil droplets, vesicles, and micelles, respectively.

### UV-visible spectroscopy to determine CAC

A stock of 1 mg/mL merocyanine 540 was prepared in 50% (vol/vol) ethanol. Various concentrations (from 0 μM to 1 mM) of lipid solutions were prepared from thin films of 1Pro-C18:1 and 2Pro-C18:1 individually in 0.2 M bicine, 50 mM NaCl, pH 8.0. 200-μL aliquots were placed in a 96-well plate followed by the addition of 1.0 μL merocyanine 540 (final concentration of merocyanine was 5 μM). Absorbance was recorded from 450 to 650 nm with the Epoch 2 BioTek microplate reader of the Microbiology Lab of the Chemistry Department facility. The intersection of the linear portions of plots of absorbance at 570 nm versus total concentration of lipid was used to determine the CACs.

### Size exclusion chromatography

Solutions of 10 mM 1Pro-C18:1 and 2Pro-C18:1 were prepared from thin lipid films as described above in 0.2 M bicine, 50 mM NaCl, 0.25 mM HPTS, pH 8.0. The samples were vortexed for 1–2 min. 50 μL of each sample was then loaded onto a Sephadex G-50 column, and fractions were collected in a 96-well plate with a FC203B Gilson fraction collector. Fluorescence was recorded with a SpectraMax i3x (Molecular Devices) in the Microbiology Lab, Chemistry Department facility, with excitation and emission at 465 nm and 535 nm, respectively.

### Zeta potential and DLS measurements

Zeta (ζ) potential measurements were performed using a NanoBrook 90Plus PALS particle size and zeta potential analyzer (Brookhaven Instruments). 20 mL aqueous solutions of 1 mM 1Pro-C18:1 and 2Pro-C18:1 were prepared in deionized water in a 50-mL beaker. NaOH was added from a 50 mM stock to the acidic lipid solution with a HI931 automatic potentiometric titrator (Hanna Instruments) until pH 11.0. During the titration, 1-mL aliquots were withdrawn at different pH values for measurements of zeta potential. To maintain a constant volume, this 1 mL was returned after measurement. Hydrodynamic size distributions were determined by dynamic light scattering (DLS) at 20 mM total lipid in 0.2 M bicine, 50 mM NaCl, pH 8.0. Samples were centrifuged, filtered through 0.2-μm filters, and incubated for 24 h at 23°C prior to measurement.

### Transmission electron microscopy measurements

Transmission electron microscopy (TEM) images were acquired using a FEI Morgagni 268 microscope operated at 80 kV and equipped with a Gatan Orius CCD camera. Images were collected using Gatan DigitalMicrograph software (version 1.81.78). For sample preparation, 15 μL of a 20 mM lipid solution (0.2 M bicine, 50 mM NaCl, pH 8.5) was deposited onto carbon-coated copper grids with a formvar support film (Ted Pella, 300 mesh, product no. 01753-F). After allowing the sample to adsorb for 3 min, excess solution was removed by blotting with filter paper. The grids were then negatively stained with 4% (w/v) uranyl acetate in distilled water and air-dried prior to imaging.

### Turbidity measurements

Samples were prepared and titrated identically as above for the measurement of zeta potential. However, here, 200-μL aliquots were removed from the 50-mL beaker, placed in a 96-well plate, and measurements were taken at 400 nm with the Epoch 2 BioTek microplate reader of the Microbiology Lab, Chemistry Department facility.

### Surface tension measurements

All experiments were performed using a commercial Langmuir trough (80 mm diameter; surface area ≈50.3 cm^2^) filled with 26 mL of 0.2M bicine, 50 mM NaCl, pH 8.0, maintained at room temperature (21°C). For the preparation of the monolayer, defined volumes of lipid stock solutions (5 mg mL^−1^ in chloroform) of 1Pro-C18:1 or 2Pro-C18:1 were carefully spread onto the air-water interface of a circular Teflon trough using a Hamilton microsyringe. After complete evaporation of the solvent, the system was allowed to equilibrate prior to measurement. Throughout the manuscript, lipid concentration is expressed as an effective concentration, defined as the total lipid added relative to the total subphase volume. For soluble or partially soluble amphiphiles, this value represents the total material present in the system and does not imply that all lipid remains confined to the air-water interface at any given time. For example, an effective concentration of 6 μM in 26 mL corresponds to a total lipid amount of 156 nmol, which would yield a nominal surface density of ∼3.1 nmol cm^−2^ if all lipid molecules were confined exclusively to the interface. Surface tension was measured using the Wilhelmy plate method (Surface Pressure Sensor Model PS4, Nima Technology). Increasing amounts of lipid solution were introduced onto the aqueous subphase, resulting in a concentration-dependent decrease in surface tension. For 1Pro-C18:1, increasing the lipid concentration reduced the surface tension from that of pure water (γ ≈ 72 mN·m^−1^) to approximately 36 mN·m^−1^ at ∼6 μM. However, the surface tension did not fully stabilize even after 1 h of equilibration, suggesting limited surface activity of the lipid.

### Vibrational sum frequency generation spectroscopy

The vSFG experiments were performed using a femtosecond Yb-based laser system (Carbide, Light Conversion) with 80 W power, 2 mJ pulse energy, 333 fs pulse duration, and a central wavelength of 1030 nm at 40 kHz with tunable repetition rate. The output was split into two parts: one directed to a second-harmonic generator (HERO, Light Conversion) to produce 515-nm visible light, which was spectrally narrowed using an ultra-narrow bandpass filter; the other directed to an optical parametric amplifier (OPA, ORPHEUS-MIR, Light Conversion) to generate broadband IR pulses from 2,000 to 5,000 nm. The broadband IR and narrowband visible laser beams were then focused and overlapped in both space and time on the sample surface to generate the sum frequency (SF) pulse. The output SF pulse from the sample surface was collimated and focused onto a spectrograph (Princeton Instruments, Acton SP-2556, 600 grooves/mm grating) connected to a thermoelectrically cooled (−75°C) charge-coupled device camera (Princeton Instruments, PIXIS 100B). The visible (∼1.75 μJ/pulse) and infrared (IR) (∼5 μJ/pulse) beams were directed at the sample cell at incident angles of 45° and 47°, respectively, relative to the surface normal. We used SSP (S-polarized SFG, S-polarized visible, and P-polarized IR) polarization combination for the measurements. The spectra were background corrected and then normalized with respect to the reference spectrum of a gold to account for any fluctuation in the energies of the incident beams and to account for the nonresonant signal. The vSFG measurements were performed on the same 8-cm-diameter Teflon trough used for surface pressure measurements. Different concentrations of lipid were added, equilibrated for ∼50 min, and then the vSFG spectra were recorded.

The VSFG signal intensity, denoted as *I*_*SFG*_, is directly proportional to the product of the square of the second-order nonlinear susceptibility tensor of the sample, *χ*^(2)^, and the intensities of both visible beams, *I*_*VIS*_, and the IR beams, *I*_*IR*_:

(Equation 1) $$I_{SFG}={\left|E_{sig}e^{i\varphi_{sig}}\right|}^{2}I_{VIS}I_{IR}\propto {\left|\chi_{total}^{\left(2\right)}\right|}^{2}\text{,}$$

where $\chi_{total}^{\left(2\right)}$ comprises a resonant and a nonresonant part,

(Equation 2) $${\left|\chi_{total}^{\left(2\right)}\right|}^{2}={\left|\chi_{NR}^{\left(2\right)}e^{i\varphi_{NR}}+\sum_{\nu }\frac{A_{\nu }e^{i\varphi_{\nu }}}{\omega_{\nu }-\omega_{IR}-i\Gamma_{\nu }}\right|}^{2}$$

Here, $\chi_{NR}^{\left(2\right)}e^{i\varphi_{NR}}$ accounts for the nonresonant second-order susceptibility and its phase. The second term represents the vibrational resonant contribution. In this expression, *A*_*ν*_, *ω*_*ν*_, and *Γ*_*ν*_ represent the resonant amplitude, resonant frequency, and line width of the *ν*^*th*^ vibrational transition, respectively. Together, these terms determine the absolute phase of the SF spectrum (*φ*_*sig*_) with the corresponding vSFG electric field magnitude or signal E_sig._ (23). Based on Equation (2), the absolute square of the sum of Lorentzian functions was used to fit the vSFG spectra of all the lipids at air-water interfaces. The fitting values can be found in Tables S1, S2, and S3. The C-H bands, located approximately at 2,850 cm^−1^, 2,880 cm^−1^, 2,926 cm^−1^, and 2,950 cm^−1^ were fit with widths ranging from 26, 28, 20, and 16 cm^−1^, respectively. Throughout the fitting process, the widths of all bands (*Γ*_*ν*_) remained constant when analyzing the data for various concentrations of lipids added to the air-water interface.

## Results and discussion

### 1Pro-C18:1 and 2Pro-C18:1 form vesicles

**Figure 1:**
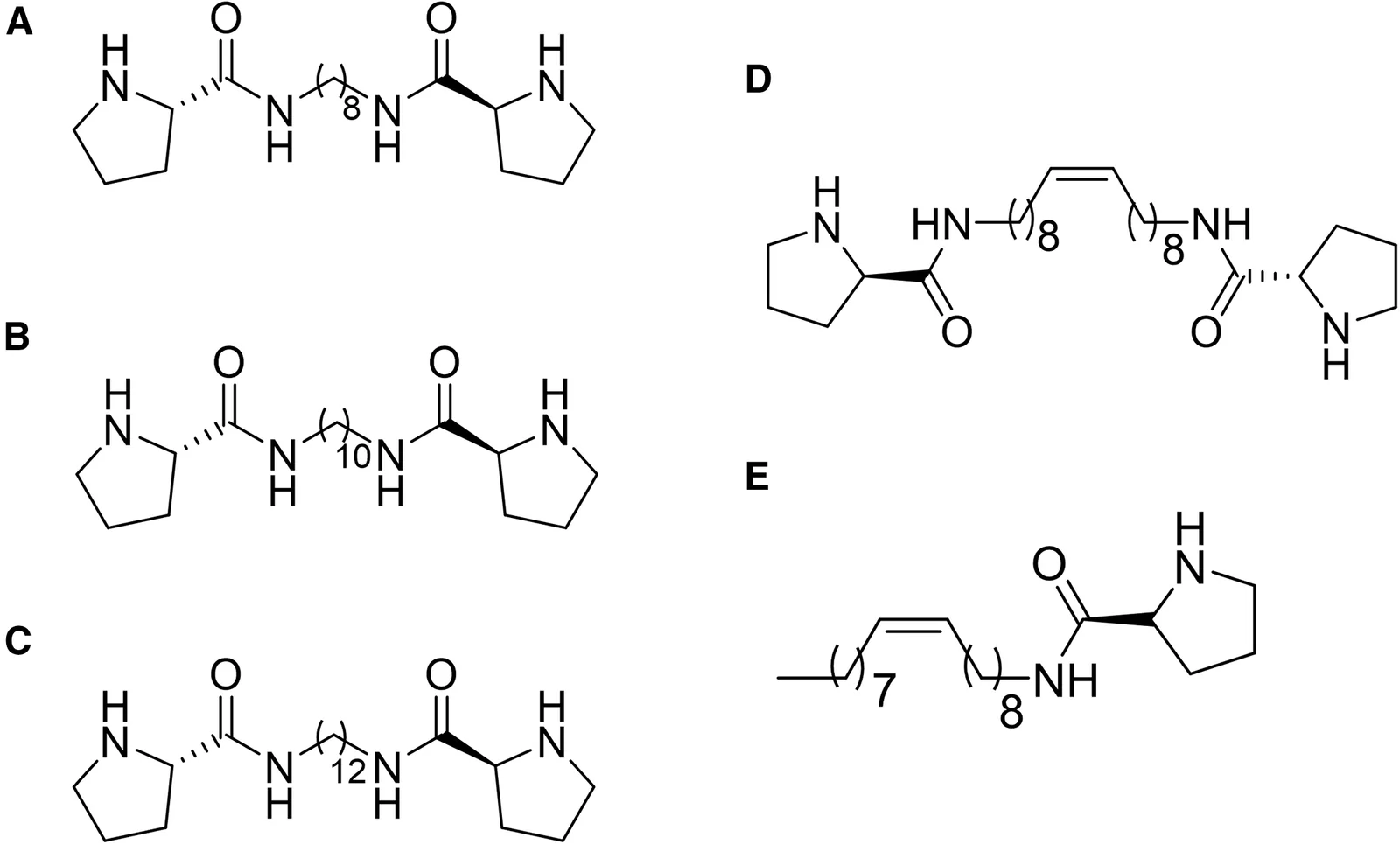
Chemical structures of synthesized lipids. (A) 2Pro-C8:0, (B) 2Pro-C10:0, (C) 2Pro-C12:0, (D) 2Pro-C18:1 (bolalipids with two proline headgroups and varying alkyl chain lengths), and (E) 1Pro-C18:1 (unipolar analog with one proline headgroup).

Single-chain bolalipids with L-proline (L-Pro) headgroups separated by 8, 10, 12, and 18 carbons were synthesized by EDC (1-ethyl-3-(3-dimethylaminopropyl)carbodiimide) coupling of amino-terminated hydrocarbons with Boc-protected L-Pro, similar to the report by Hu et al. (25). As a result of this linking strategy, the L-Pro group can be positive (at lower pH) or neutral (at higher pH). We refer to these lipids here as 2Pro-C8:0, 2Pro-C10:0, 2Pro-C12:0, and 2Pro-C18:1, respectively (Fig. 1 *A*–*D*). The lipids shown in Fig. 1 *A*–*C* were saturated, while 2Pro-C18:1 contained a *cis*-double bond at C9 of the hydrocarbon chain (Fig. 1 *D*). A unipolar lipid analog, 1Pro-C18:1, containing only one Pro headgroup, was synthesized similarly (Fig. 1 *E*). All lipids were purified by silica column chromatography and the structures confirmed by ^1^H and ^13^C NMR spectroscopy (Figs. S1, S2, S3, S4, and S5).

**Figure 2:**
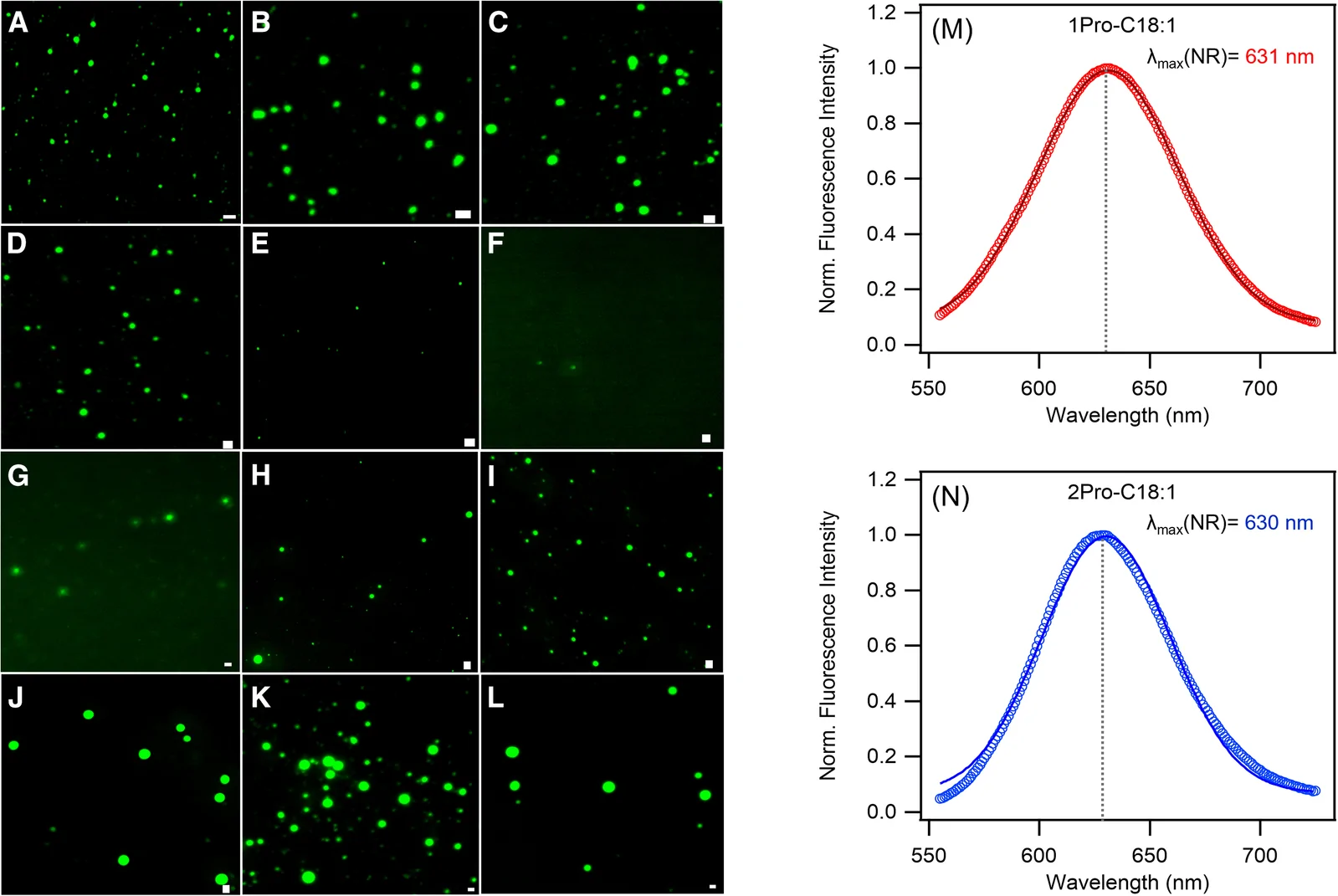
pH-dependent formation of 1Pro-C18:1 and 2Pro-C18:1 lipid vesicles. Confocal fluorescence microscopy images of vesicles formed by 1Pro-C18:1 (A–F) and 2Pro-C18:1 (G–L) at pH 6.0 (A, G), pH 7.0 (B, H), pH 7.5 (C, I), pH 8.0 (D, J), pH 8.5 (E, K), and pH 9.0 (F, L). The scale bar represents 5 μm. Nile Red (NR) fluorescence emission spectra for 1Pro-C18:1 (M) and 2Pro-C18:1 (N) confirm the formation of a vesicular membrane with characteristic emission maxima near 630 nm.

Thin lipid films were prepared by dissolving each lipid in 1:1 chloroform:methanol followed by rotary evaporation. The films were hydrated with buffered aqueous solutions (pH 6.0 to 9.0) containing 0.25 mM HPTS (8-hydroxypyrene-1,3,6-trisulfonic acid), a hydrophilic fluorophore, and the resulting structures were evaluated by confocal fluorescence microscopy. At 20 mM total lipid, no aggregates were observed for 2Pro-C8:0 and 2Pro-C10:0, and only a small number of nonspherical aggregates were detected for 2Pro-C12:0 (Fig. S6). While higher concentrations were not systematically explored, shorter hydrophobic chains are generally expected to reduce aggregate stability and favor micellar or nonvesicular structures. In contrast, 1Pro-C18:1 clearly formed vesicles between pH 6.0 and 8.0 but not at higher pH (Fig. 2 *A*–*F*). Similarly, 2Pro-C18:1 formed well-defined spherical vesicles between pH 7.0 and 9.0 (Fig. 2 *G*–*L*). Because imaging was performed after dilution, the data were consistent with the formation of vesicles capable of retaining a small (524.39 g/mol) hydrophilic fluorophore. TEM images further supported vesicular morphology rather than micellar aggregates for both 1Pro-C18:1 and 2Pro-C18:1 (Fig. S9 *C*, *D*), although bilayer thickness could not be resolved under the present imaging conditions. Spontaneous vesicle formation upon simple hydration is consistent with the behavior of many single-chain amphiphiles (26), which typically exhibit lower energetic barriers to membrane curvature than conventional diacyl phospholipids. However, such behavior is less well documented for single-chain bolalipids, which are often associated with increased membrane rigidity.

To further confirm that the observed spherical aggregates were lipid vesicles, the samples were probed by fluorescence spectroscopy with Nile Red. The fluorophore Nile Red is a lipophilic, solvatochromic dye that exhibits characteristic emission near 630 nm when partitioned into vesicular membranes and near 640 nm when associated with micelles (27). Upon incubation with 10 mM 1Pro-C18:1 and 2Pro-C18:1, emission maxima at 631 nm (Fig. 2 *M*) and 630 nm (Fig. 2 *N*), respectively, were observed, consistent with vesicular membrane environments. DLS measurements revealed nanoscale assemblies with average hydrodynamic diameters of ∼130 nm for 1Pro-C18:1 and ∼340 nm for 2Pro-C18:1 (Fig. S9), consistent with vesicle-sized aggregates. The coexistence of nanoscale vesicles detected by DLS and larger structures observed by confocal microscopy likely reflects sample polydispersity, with DLS reporting an intensity-weighted size distribution and optical microscopy visualizing larger, optically resolvable vesicles.

The selective formation of vesicles observed for the C18:1 system likely reflects a combination of hydrophobic length and chain fluidity. The shorter bolalipids (C8–C12) may lack sufficient hydrophobic surface area to stabilize extended membrane structures in this single-chain architecture. In contrast to conventional bilayers formed by lipids with a single headgroup, a fully extended single-chain bolalipid would generate a monolayer-type membrane, requiring a longer hydrophobic segment to achieve comparable stabilization. The C18 chain, therefore, appears to provide a threshold hydrophobic length for robust vesicle formation under the present conditions. The presence of a *cis*-double bond further lowers the melting temperature and increases chain fluidity, which may facilitate self-assembly and dynamic exchange; however, because a saturated C18 analog was not examined, the relative contributions of chain length and unsaturation cannot be definitively separated here.

### 1Pro-C18:1 and 2Pro-C18:1 have a similar propensity to form vesicles

**Figure 3:**
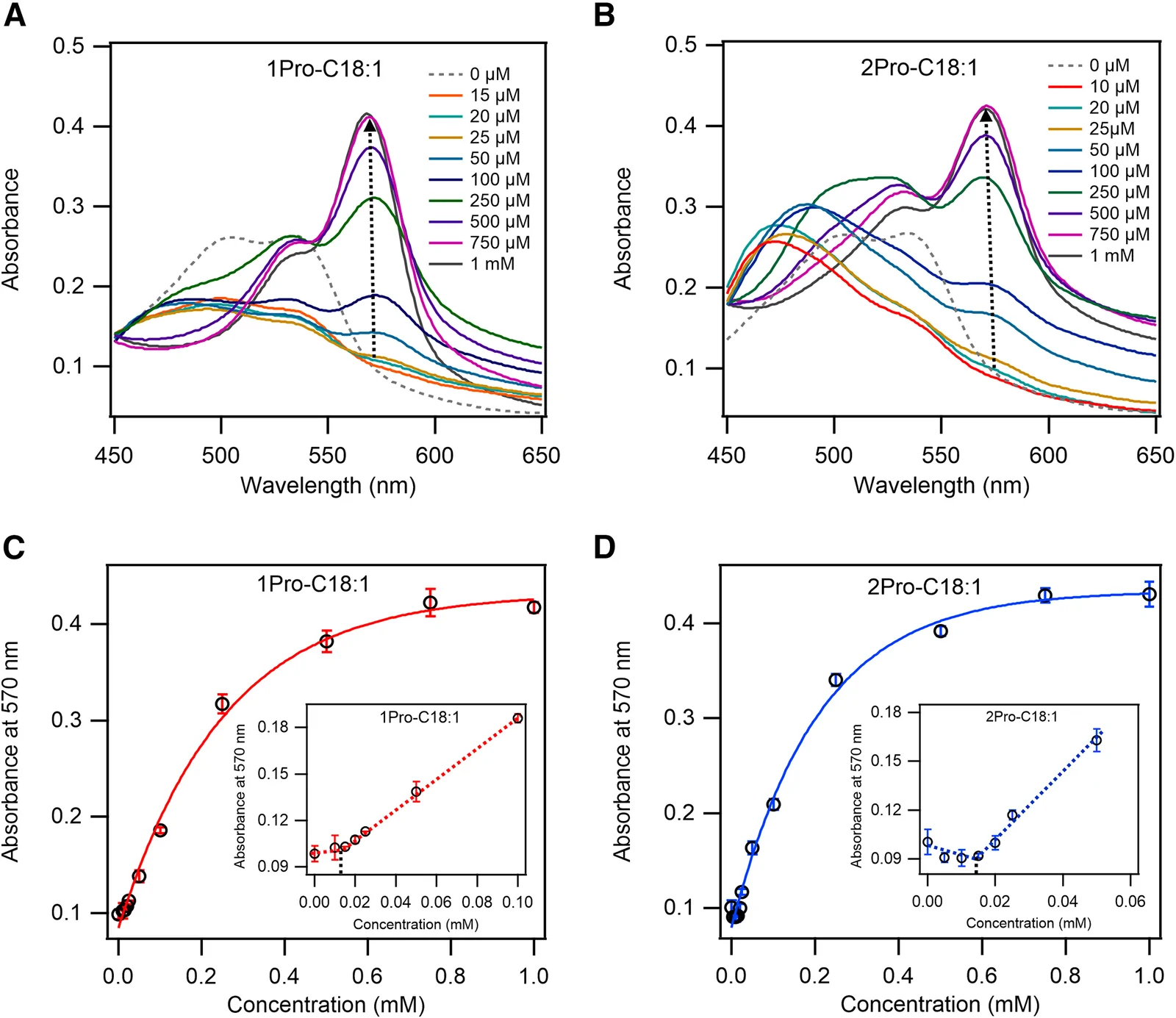
Determination of the CAC with merocyanine 540. Absorbance spectra of merocyanine 540 as a function of lipid concentration for (A) 1Pro-C18:1 and (B) 2Pro-C18:1 at pH 8.0. The emergence of a peak near 570 nm indicates the formation of vesicles. (C and D) Absorbance versus concentration plot to determine the critical aggregation concentration. Error bars represent ± standard deviation (SD); *n* = 2.

To assess the thermodynamic favorability of the formation of vesicles, the CAC of 1Pro-C18:1 and 2Pro-C18:1 was determined using merocyanine 540. In the absence of vesicles, merocyanine 540 exhibits absorbance maxima near 500 and 540 nm, corresponding to dimeric and monomeric forms of the fluorophore in water, respectively, (Fig. 3 *A*, *B*) (28). Upon addition of either 1Pro-C18:1 or 2Pro-C18:1, an absorbance peak near 570 nm emerged, characteristic of the presence of vesicles (28). Fitting of absorbance as a function of the concentration of lipid yielded CAC values of 18 μM ± 2 μM for 1Pro-C18:1 and 19 μM ± 2 μM for 2Pro-C18:1 at pH 8.0 (Fig. 3 *C*, *D*, inset). The similarity of these CAC values indicates that the presence of two proline headgroups in the bolalipid does not substantially alter the thermodynamic driving force for aggregation relative to the unipolar lipid. Moreover, the CAC values are comparable to that reported for the C18:1 fatty acid oleate (29), suggesting that aggregation in this system is largely governed by the hydrophobic C18 chain rather than by the charge of the headgroup.

This result is somewhat counterintuitive. A membrane-spanning bipolar lipid might be expected to exhibit enhanced rigidity or a stronger thermodynamic driving force for vesicle formation relative to a unipolar analog, analogous in some respects to archaeal tetraether lipids that form highly stable monolayer membranes. However, the comparable CAC values observed here suggest that aggregation thermodynamics are largely dictated by hydrophobic chain contributions with headgroup architecture exerting a more subtle effect. One possible explanation is that 2Pro-C18:1 does not adopt a fully extended membrane-spanning conformation in bulk vesicles but instead may assume a bent or U-shaped configuration, thereby reducing the effective architectural distinction between the uni- and bipolar lipids. While the interfacial vSFG data below are consistent with such a bent conformation at the air-water interface, the conformation adopted within vesicles remains uncertain and will require future structural investigation.

### 1Pro-C18:1 and 2Pro-C18:1 form stable vesicles

To confirm the stability of the vesicles and the ability to encapsulate material, size exclusion chromatography was performed on vesicles loaded with the fluorophore HPTS. Given the prior evidence that fluorescence microscopy can be used to observe vesicles that are insufficiently stable to survive size exclusion chromatography (30), it is important to corroborate the presence of vesicles by several techniques. For both 1Pro-C18:1 and 2Pro-C18:1, two fluorescent elution peaks containing HPTS were observed, with the first peak corresponding to vesicles with entrapped HPTS and the second peak reflecting free, unentrapped HPTS. The chromatograms clearly showed that both 1Pro-C18:1 (Fig. S8A) and 2Pro-C18:1 (Fig. S8B) formed vesicles. Importantly, the vesicles persisted in the presence of monovalent and divalent cations, conditions that are more disruptive for fatty acid vesicles (31). Specifically, vesicles consisting of either 1Pro-C18:1 or 2Pro-C18:1 were stable in the presence of 250 mM NaCl, 250 mM KCl, 10 mM Mg^2+^, and 10 mM Ca^2+^ at pH 7.5 (Fig. S7A–S7H). Stabilization was likely due to weaker interactions between the cations and the Pro headgroup. While the present study establishes vesicle formation and stability, additional characterization using environment-sensitive probes could further clarify membrane packing and hydration properties relative to conventional bilayers.

### Partitioning to membranes shifts the apparent pK_a_ of the Pro headgroup

**Figure 4:**
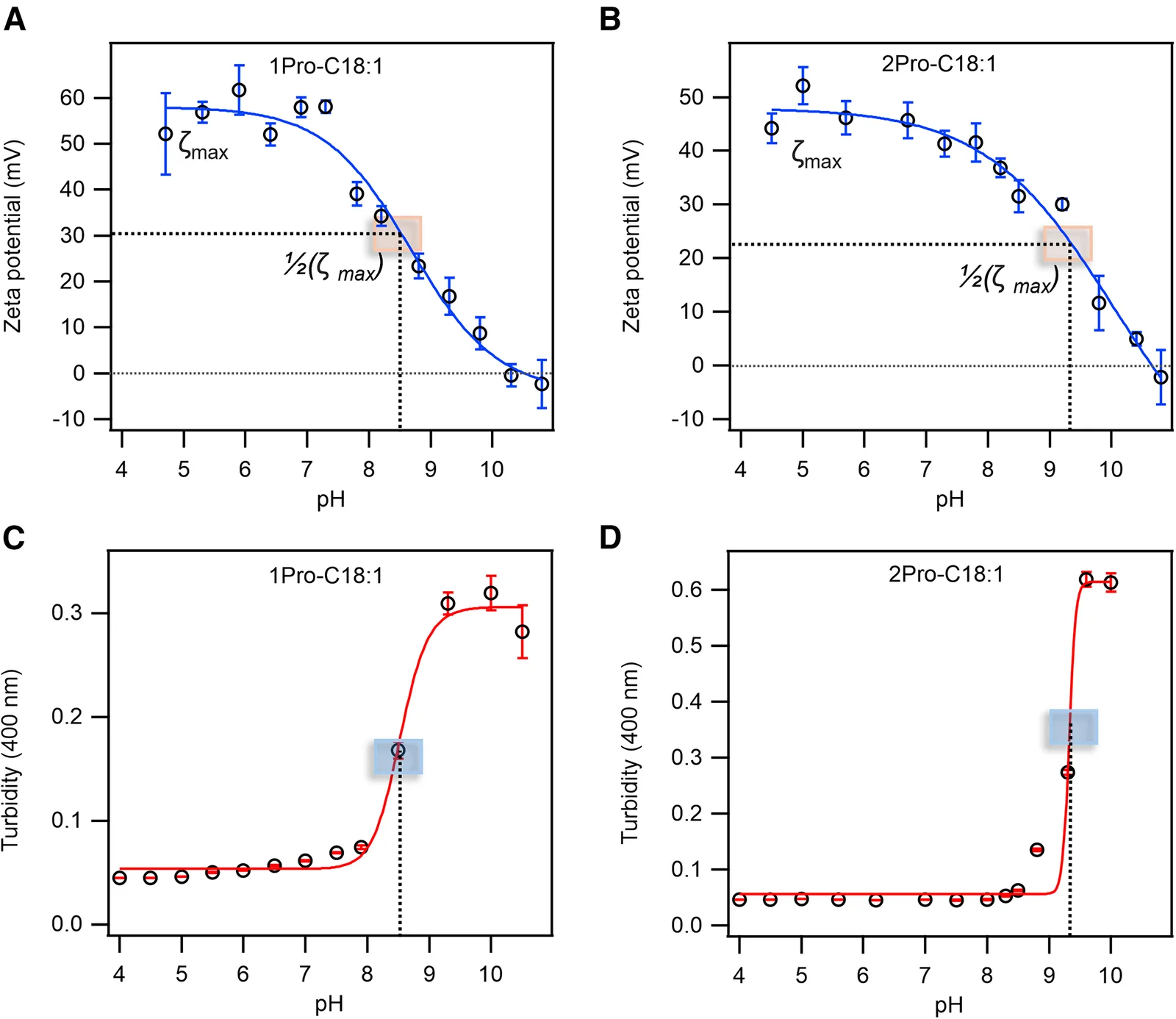
Correlation between ζ potential, turbidity, and pK_a_ for 1Pro-C18:1 and 2Pro-C18:1. (A and B) ζ potential measurements across pH 6–11, showing a transition from positive surface charge to neutral charge. (C and D) Corresponding turbidity measurements, where increased scattering correlates with vesicle formation at higher pH. Estimated pK_a_ values are indicated. Error bars represent ± standard deviation (SD); *n* = 3 (*A* and *B*), *n* = 2 (*C* and *D*).

To probe the ionization behavior of the lipids within membranes, we measured the zeta (ζ) potential of vesicles as a function of pH. The ζ potential was determined by electrophoretic light scattering, in which an applied electric field induces vesicle migration, and electrophoretic mobility is obtained from the Doppler shift of scattered light. The protonated amine-heterocycle of free proline has a reported pK_a_ of 10.6 (32). Because functional groups can exhibit shifted pK_a_ values upon partitioning into a membrane (33,34), the pH dependence of the ζ potential was used to estimate the apparent pK_a_ of 1Pro-C18:1 and 2Pro-C18:1 in vesicle membranes. Since the ζ potential reflects the electrostatic potential at the slipping plane of intact vesicles, measurements of ζ potential report on the net surface charge arising from ionized headgroups in the membrane. Free monomers in solution do not contribute detectably to the measured signal under these conditions. The ζ potentials of both 1Pro-C18:1 and 2Pro-C18:1 were greater than approximately +30 mV at pH 6.0–7.0 (Figs. 4 *A*, *B*), consistent with the formation of a stable dispersion of positively charged aggregates. Increasing pH led to a progressive decrease in ζ potential, which approached 0 mV between pH 10 and 11. The apparent pK_a_ was estimated by fitting the ζ potential versus pH data to a sigmoidal function and extracting the midpoint of the transition, as previously described (33), yielding values of 8.5 ± 0.2 and 9.3 ± 0.1 for 1Pro-C18:1 and 2Pro-C18:1, respectively.

To corroborate these values, turbidity was recorded as a function of pH under similar conditions (Figs. 4 *C*, *D*). Turbidity increased with increasing pH, with inflection points at 8.4 ± 0.1 and 9.2 ± 0.1 for 1Pro-C18:1 and 2Pro-C18:1, respectively, closely matching the apparent pK_a_ values derived from the ζ potential measurements. The correspondence between the ζ potential transition and the turbidity inflection indicates that the protonation state of the lipid is coupled to aggregate structure. At low pH, protonated lipids generate strong interfacial charge that limits the formation of larger light-scattering assemblies. As the pH increases and the lipids become progressively deprotonated, surface charge decreases and larger aggregates are formed, reflected by the increase in turbidity. The apparent pK_a_ values are lower than that of free proline, indicating differential stabilization of the protonation states upon incorporation into the membrane. This downward shift is consistent with relative stabilization of the neutral form and/or destabilization of the protonated form within the aggregate, thereby shifting the protonation equilibrium to lower pH.

The proline headgroup introduces a stereocenter (L-Pro), raising the question of whether inverted or mixed chirality (D-Pro or D/L mixtures) would alter membrane behavior. In the absence of other chiral interaction partners, enantiomeric amphiphiles are generally expected to exhibit similar bulk thermodynamic self-assembly properties, and thus the apparent pK_a_ inferred from interfacial charge would likely be comparable for D- and L-Pro variants. However, membrane chirality can become functionally relevant through subtle differences in interfacial organization and chiral recognition phenomena. For example, chiral phospholipid bilayers can exhibit enantioselective permeation of amino acid derivatives (35), stereoselective interactions with membrane-active peptides (36), and differences in collective organization between enantiopure and racemic lipid assemblies under certain conditions (37). Moreover, systematic modulation of lipid chirality in minimal living membranes has been shown to affect cellular fitness and membrane behavior, underscoring the broader biological relevance of membrane chirality (38). Future work could therefore test whether D-Pro variants or mixed-chirality Pro-lipids produce measurable differences in interfacial charge, permeability, or molecular recognition.

### Limited surface activity of 1Pro-C18:1 and 2Pro-C18:1

**Figure 5:**
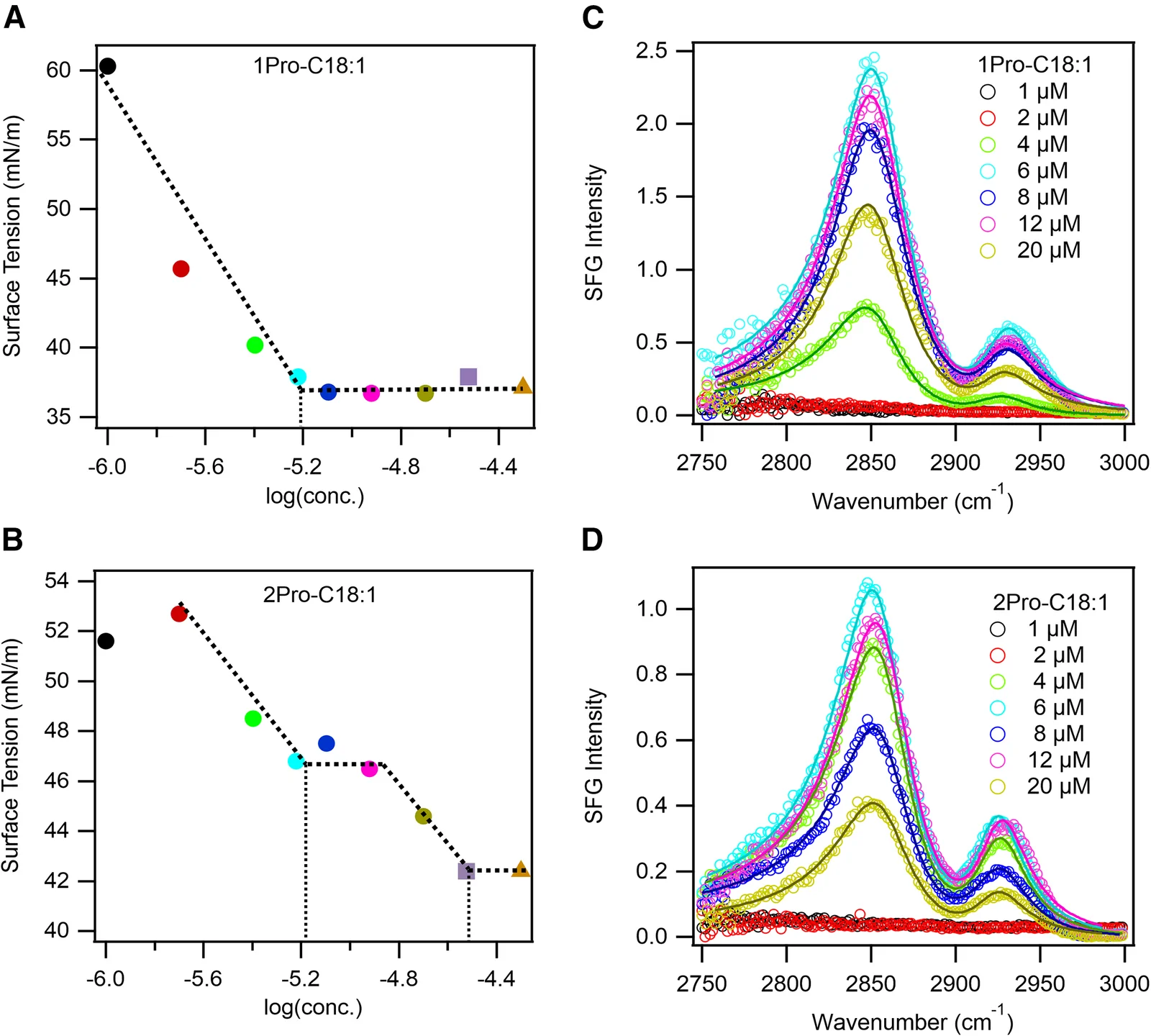
Surface tension and vSFG analysis of 1Pro-C18:1 and 2Pro-C18:1 at the air-water interface. Surface tension as a function of lipid concentration for 1Pro-C18:1 (A) and 2Pro-C18:1 (B) measured in buffer (pH 8.0, 50 mM NaCl). Inflection points in the surface tension isotherms are indicated. vSFG spectra of 1Pro-C18:1 (C) and 2Pro-C18:1 (D) at varying concentrations under identical buffer conditions. Spectra were referenced to allow comparison of relative intensities across experiments. Complete fitting parameters are provided in Tables S1 and S2. All vSFG measurements were recorded after ∼50 min of equilibration.

Although the aggregation behavior of both lipids in bulk solution was similar, we hypothesized that interfacial assembly would be more sensitive to molecular architecture. To probe this behavior, we measured surface tension as a function of lipid concentration at the air-water interface (Figs. 5 *A*, *B*). At 1 μM, both lipids reduced the surface tension of pure water (from ∼72 mN/m to ∼60 and 52 mN/m for 1Pro-C18:1 and 2Pro-C18:1, respectively), confirming significant surface activity. The concentration-dependent evolution of surface tension, however, differed from that of classical soluble C18:1 surfactants. Under identical conditions, oleate and oleylamine exhibited surface tension plateaus at concentrations that closely coincided with their CACs (Fig. S10), consistent with the expected coupling between interfacial adsorption and bulk aggregation for soluble surfactants. In such systems, once bulk aggregation begins, the monomer activity, and therefore the interfacial surface excess, remains approximately constant. In contrast, for 1Pro-C18:1 and 2Pro-C18:1, the concentration at which surface tension exhibited a clear change in slope near 6 μM did not coincide with the CAC determined from bulk measurements (∼18–20 μM) (Figs. 5 *A*, *B*). This separation indicated that interfacial adsorption and bulk aggregation were not simply coupled in these systems. Instead, adsorption to the interface and formation of bulk aggregates competed over a broad concentration range. We therefore interpret the first transition (∼6 μM) as primarily interfacial in origin, whereas the second transition observed for 2Pro-C18:1 likely reflects the increasing influence of bulk aggregation on monomer activity and interfacial coverage.

### Symmetry-dependent interfacial conformations

To further gain molecular-level insight into the distinct behavior of unipolar 1Pro-C18:1 and bipolar 2Pro-C18:1 lipids, we used vSFG spectroscopy, which is highly sensitive to molecular order and orientation at interfaces (23,39). Experimental details for the vSFG setup are provided in the supplemental information. Figs. 5 *C*, *D* present the vSFG spectra of the air-water interface in the presence of 1Pro-C18:1 and 2Pro-C18:1, respectively. The interface in the presence of lipid 1Pro-C18:1 was characterized by peaks at ∼2,852 cm^−1^ and ∼2,926 cm^−1^, corresponding to the symmetric stretching (CH_2_^ss^) and asymmetric stretching vibrations (CH_2_^as^), respectively, of the hydrocarbon chains of these lipids (40). The absence of distinct CH_3_ symmetric stretch and Fermi resonance features in the ssp spectra of 1Pro- C18:1 reflects the limited spectral resolution of our vSFG setup (∼39 cm^−1^). Under these conditions, the expected –CH_3_ modes (∼2,875–2,880 cm^−1^ and ∼2,940 cm^−1^) overlap substantially with the more intense –CH_2_ symmetric and antisymmetric stretches, producing a broadened envelope dominated by methylene contributions. Resolving individual –CH_3_ features would require significantly higher spectral resolution (≈10–15 cm^−1^) than available in the present configuration.

**Figure 6:**
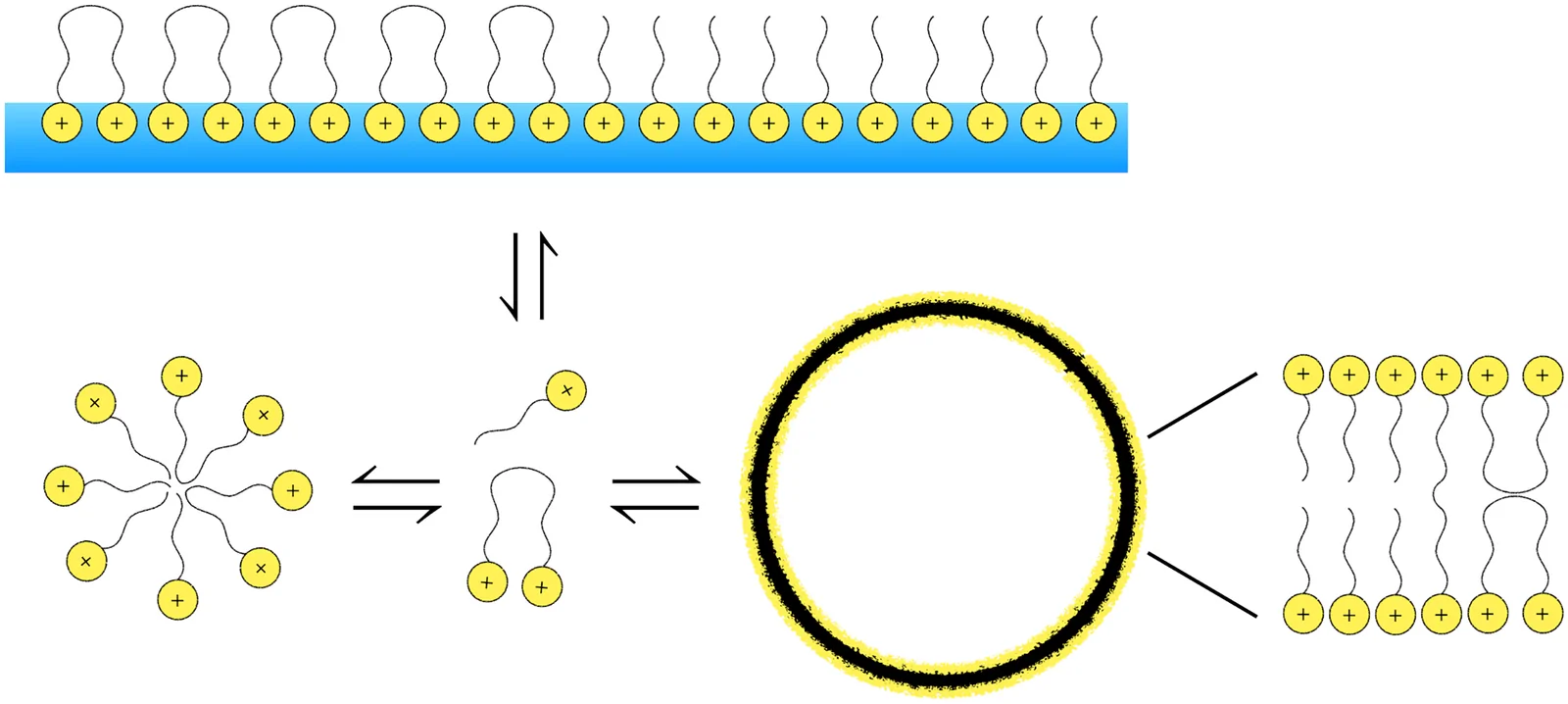
Schematic representation of the dynamic exchange between interfacial and bulk-associated lipid species. At low effective concentrations (4–6 μM), lipids adsorb to the air-water interface while remaining in equilibrium with bulk monomer. At higher effective concentrations, additional aggregation states (e.g., micelle-like or vesicular assemblies) coexist with interfacial lipid. The scheme illustrates the dominant interfacial organization under high-coverage (near-saturation) conditions and is not intended to depict the instantaneous distribution of all lipid molecules in the system.

In contrast, 2Pro-C18:1 at the interface showed a ∼4 cm^−1^ blue shift in the CH_2_ symmetric stretch (supplemental information, Tables S1 and S2) and the appearance of an additional peak at ∼3,010 cm^−1^ (Fig. S11B). The latter feature corresponds to the alkene C–H stretch; absence of this peak in spectra of 1Pro-C18:1 (Fig. S11A) suggested an altered conformation at the interface, such as a U-shaped (or “horseshoe”) conformation (Fig. 6) for 2Pro-C18:1 (41,42). In this U-shaped conformation, both proline headgroups are expected to remain solvated in the aqueous phase, while the unsaturated hydrocarbon chain extends into air. The presence of a *cis*-double bond may introduce conformational constraints, thereby favoring such a bent interfacial geometry (42). A saturated C18:0 analog would likely exhibit reduced conformational flexibility and a higher melting temperature, potentially favoring more ordered interfacial packing and altering the balance between extended and bent geometries. Because such an analog was not examined in the present study, the specific role of unsaturation in stabilizing the proposed U-shaped conformation remains to be determined. By comparison, 1Pro-C18:1 favored an extended geometry that resulted in suppression of this SFG feature.

The vSFG measurements at the air-water interface of 1Pro-C18:1 in buffered water were very slow to stabilize after evaporation of the organic solvent used to deposit the lipid. This longer equilibration time is consistent with the surface tension measurements and indicates equilibrium between the interface and bulk solution. Unlike diacyl phospholipids, which form kinetically trapped aggregates, single-chain lipids equilibrate between different structures (15). The equilibration process was observed by the gradual decay of the vSFG intensity of the CH_2_ peak over time (Fig. S12). Stabilization of the signal was achieved only after ∼50 min. Therefore, all subsequent vSFG spectra were recorded after a 50-min equilibration period.

Varying the lipid concentration revealed a nonmonotonic dependence of the vSFG signal for 1Pro-C18:1, with the CH_2_ vibrational intensity increasing from 4 μM to 6 μM before decreasing at higher concentrations (Figs. 5 *C*). The increased intensity of both symmetric (lower wavenumber) and antisymmetric (higher wavenumber) CH_2_ stretching vibrations from 4 μM to 6 μM is consistent with a greater number of monomers adsorbed at the interface with increasing concentration. These data also agreed with the surface tension measurements, where the surface tension exhibited a clear change in slope near 6 μM. However, beyond this point, the intensity of both CH_2_ vibrations decreased with increasing concentration, with a significant drop at 20 μM. This decreased signal was consistent with increased partitioning of lipid into bulk aggregates. At low concentrations, 1Pro-C18:1 exists in dynamic exchange between the air-water interface and monomer in bulk solution. At higher concentrations, additional equilibria arise between 1Pro-C18:1 at the air-water interface, free monomer in solution, and lipid embedded within bulk-associated aggregates (e.g., micelle-like or vesicular species). Similar equilibria between the air-water interface and vesicles in solution were previously reported (12,43,44).

2Pro-C18:1 behaved similarly. At concentrations below 4 μM, distinct CH_2_ vibrational peaks appeared in the vSFG spectra initially but gradually diminished over time and eventually disappeared with increasing equilibration time, indicating progressive equilibration of lipid molecules with the bulk phase (Fig. S13). At 6 μM, the C-H stretching vibrational intensity of the CH_2_ vibration reached a maximum, consistent with surface tension measurements where the first inflection was observed. With increasing concentration, the intensity of the CH_2_ vibrational peaks varied nonmonotonically (Figs. 5 *D*), suggesting changes in interfacial configuration or the existence of equilibria between interfacial, free, and aggregated 2Pro-C18:1. The data suggest lower surface activity for 2Pro-C18:1 relative to 1Pro-C18:1. This is supported by two key observations. First, 2Pro-C18:1 induced a smaller overall decrease in surface tension, and second, 2Pro-C18:1 produced a significantly lower SFG intensity (more than 2-fold lower than 1Pro-C18:1 at 6 μM). The complexity of the assembly of 2Pro-C18:1 is further highlighted by the nonmonotonic variation in SFG intensity exhibited by this lipid near the CAC. Such behavior is consistent with multiple concentration-dependent equilibria between interfacial and bulk-associated species.

As a reference for an insoluble phospholipid monolayer, we performed concentration-dependent vSFG measurements on dipalmitoylphosphatidylcholine (DPPC), a lipid known to form a stable interfacial monolayer (Fig. S14). As expected, DPPC exhibited a monotonic increase in CH_2_ intensity with concentration, and at higher surface coverage, the monolayer transitioned into a liquid-condensed phase, accompanied by characteristic changes in the vSFG line shape and appearance of the symmetric stretch of CH_3_ vibrations at ∼2,880 cm^−1^ (39). In contrast, neither 1Pro-C18:1 nor 2Pro-C18:1 showed such spectral changes, suggesting that the behavior observed above 6 μM was not due to changes in packing. Taken together, these observations are consistent with a concentration-dependent shift in equilibrium toward bulk-associated aggregates.

To further probe possible conformational changes in the alkyl chains of 1Pro-C18:1 and 2Pro-C18:1, we calculated the ratio of amplitude of antisymmetric (ν_as_) and symmetric (ν_s_) methylene stretching modes $\left(A_{CH_{2}^{as}}/A_{CH_{2}^{ss}}\right)$. The frequencies of the antisymmetric (ν_as_) and symmetric (ν_s_) methylene stretching modes are sensitive to chain conformation and can be empirically correlated with the *trans*/*gauche* ratio of the alkyl chains (41). The amplitude ratio showed no significant variation with increasing concentration for either 1Pro-C18:1 (Table S1, 0.22 ± 0.05) or 2Pro-C18:1 (Table S2, 0.35 ± 0.04). These results are consistent with reduced interfacial population rather than changes in alkyl chain conformation.

Fig. 6 illustrates the proposed interfacial organization of these lipids at the air-water (buffer) interface based on surface tension, vSFG, and bulk aggregation measurements. At low concentrations (∼4 μM), both lipids exhibit dynamic exchange between the air-water interface and bulk monomer. Above ∼6 μM, concentration-dependent equilibria involving bulk-associated aggregates increasingly influence the distribution of lipid between the interface and solution. The balance between interfacial adsorption and bulk aggregation differs for 1Pro-C18:1 and 2Pro-C18:1, with 2Pro-C18:1 exhibiting reduced interfacial population at higher concentrations.

### Conclusion

Unipolar 1Pro-C18:1 and bipolar 2Pro-C18:1 lipids exhibit remarkably similar bulk self-assembly behavior. Both form vesicles over comparable ranges of pH with similar stability, and both display similar CACs, comparable to that of the C18:1 fatty acid oleate (29). The differences observed in solution primarily reflect differences in the apparent pK_a_ of the Pro headgroup between the uni- and bipolar lipids. Together, these results suggest that aggregation thermodynamics in this system are governed largely by the hydrophobic C18 chain, while headgroup architecture exerts a more subtle influence.

Single-chain lipids are generally more dynamic than their double-chain counterparts (45), and this dynamic character was evident at the air-water interface. Both lipids exhibited concentration-dependent redistribution between the interface and bulk-associated aggregates, with reduced interfacial signal observed at higher concentrations. A key distinction between the two lipids was their inferred interfacial conformation, reflected in differences in the CH_2_ symmetric stretch in vSFG spectra. The vSFG results are consistent with an extended interfacial geometry for 1Pro-C18:1 in which the headgroup resides in the aqueous phase and the hydrophobic chain extends toward air. In contrast, the spectra for 2Pro-C18:1 support a U-shaped conformation in which both headgroups remain solvated. Such a conformation may influence interfacial curvature and stabilization of dispersed phases, including aqueous aerosols (46) or water-in-oil emulsions (47). The conformation adopted by 2Pro-C18:1 within vesicles remains uncertain. 2Pro-C18:1 may assume an extended membrane-spanning conformation, forming a monolayer lipid membrane, or 2Pro-C18:1 could remain in a U-shaped arrangement within a bilayer-like structure. Resolving this question will require higher-resolution structural and kinetic studies. Overall, these findings provide insight into how molecular symmetry and headgroup architecture modulate the interfacial and bulk behavior of single-chain bolalipids and may inform the rational design of dynamic amphiphilic systems for applications ranging from drug delivery to artificial cells.

## Data and code availability

Raw data have been deposited at Zenodo and can be downloaded at https://doi.org/10.5281/zenodo.18749239.

## Acknowledgments

S.S.M. acknowledges support from the 10.13039/501100008385Italian Cystic Fibrosis Research Foundation (GenDel-CF), the 10.13039/100000879Alfred P. Sloan Foundation (G-2022-19518), and 10.13039/100000936Gordon and Betty Moore Foundation (11479). J.M.G. acknowledges the Collaborative Ultrafast Spectroscopy Laboratory (CUSL) supported by the Canada Foundation for Innovation John R. Evans Leaders Fund. We thank Dr. K. Norton (microscopy facility, Department of Biological Sciences, University of Alberta) for TEM images.

## Author contributions

P.G., H.V., B.H., C.J., J.S., H.K., and S.K. performed research; F.G.W., J.M.G., and S.S.M supervised the research; P.G. and S.S.M wrote the original draft of the manuscript. All authors reviewed and edited the manuscript.

## Declaration of interests

H.V., C.J., F.G.W., and S.S.M. are inventors on a patent application related to the lipid molecules described in this work.

## Declaration of generative AI and AI-assisted technologies in the writing process

During the preparation of this work, S.S.M. used ChatGPT 5.2 to improve the consistency of the wording of the final draft. After using this tool, the authors reviewed and edited the content as needed and take full responsibility for the content of the published article.

## Supplemental information

Document S1. Figures S1–S14, Tables S1–S3, and supplemental methods

Document S2. Article plus supporting material

## References

[1] Wang C., Zhang Y., Dong Y. (2021). Lipid Nanoparticle–mRNA Formulations for Therapeutic Applications. Acc. Chem. Res..

[2] Herrmann I.K., Wood M.J.A., Fuhrmann G. (2021). Extracellular vesicles as a next-generation drug delivery platform. Nat. Nanotechnol..

[3] Ji P., Harjung A., Devaraj N.K. (2025). Photochemical synthesis of natural lipids in artificial and living cells. Nat. Commun..

[4] Toparlak Ö.D., Zasso J., Mansy S.S. (2020). Artificial cells drive neural differentiation. Sci. Adv..

[5] Chen I.A., Walde P. (2010). From self-assembled vesicles to protocells. Cold Spring Harb. Perspect. Biol..

[6] Toparlak Ö.D., Sebastianelli L., Mansy S.S. (2023). Cyclophospholipids Enable a Protocellular Life Cycle. ACS Nano.

[7] Thaipurayil Madanan K., Li Y., Bonfio C. (2024). Mg2+-driven selection of natural phosphatidic acids in primitive membranes. Chem. Sci..

[8] Goers R., Thoma J., Meier W. (2018). Optimized reconstitution of membrane proteins into synthetic membranes. Commun. Chem..

[9] Hanford M.J., Peeples T.L. (2002). Archaeal tetraether lipids. Appl. Biochem. Biotechnol..

[10] De Rosa M., Gambacorta A., Gliozzi A. (1986). Structure, biosynthesis, and physicochemical properties of archaebacterial lipids. Microbiol. Rev..

[11] Gambacorta A., Gliozzi A., De Rosa M. (1995). Archaeal lipids and their biotechnological applications. World J. Microbiol. Biotechnol..

[12] Chen S., Costil R., Feringa B.L. (2021). Self-Assembly of Photoresponsive Molecular Amphiphiles in Aqueous Media. Angew. Chem. Int. Ed. Engl..

[13] Mansy S.S. (2010). Membrane transport in primitive cells. Cold Spring Harb. Perspect. Biol..

[14] Gebicki J.M., Hicks M. (1976). Preparation and properties of vesicles enclosed by fatty acid membranes. Chem. Phys. Lipids.

[15] Luisi P.L. (2001). Are Micelles and Vesicles Chemical Equilibrium Systems?. J. Chem. Educ..

[16] Liang K., Hui Y. (1992). Vesicle of hybrid bolaamphiphile: flip-flop behavior of spin labels. J. Am. Chem. Soc..

[17] Köhler K., Förster G., Blume A. (2004). Self-Assembly in a Bipolar Phosphocholine–Water System: The Formation of Nanofibers and Hydrogels. Angew. Chemie Int. Ed..

[18] Ambrosi M., Fratini E., Baglioni P. (2006). Nanotubes from a Vitamin C-Based Bolaamphiphile. J. Am. Chem. Soc..

[19] Lee E., Huang Z., Lee M. (2008). Rigid–Flexible Block Molecules Based on a Laterally Extended Aromatic Segment: Hierarchical Assembly into Single Fibers, Flat Ribbons, and Twisted Ribbons. Chem. Eur J..

[20] Köhler K., Förster G., Blume A. (2004). Temperature-Dependent Behavior of a Symmetric Long-Chain Bolaamphiphile with Phosphocholine Headgroups in Water: From Hydrogel to Nanoparticles. J. Am. Chem. Soc..

[21] Blume A., Drescher S., Dobner B. (2013). Tuning the aggregation behaviour of single-chain bolaphospholipids in aqueous suspension: from nanoparticles to nanofibres to lamellar phases. Faraday Discuss..

[22] Dehsorkhi A., Castelletto V., Hamley I.W. (2014). Self-assembling amphiphilic peptides. J. Pept. Sci..

[23] Zhu X.D., Suhr H., Shen Y.R. (1987). Surface vibrational spectroscopy by infrared-visible sum frequency generation. Phys. Rev. B.

[24] Gahtori P., Mishra A., Pandey R. (2023). Unravelling the Mechanism behind Charge Reversal at Silica Nanoparticle–Model Cell Membrane Interfaces. J. Phys. Chem. B.

[25] Hu B.-B., Yuan Y., Li S.-M. (2016). Synthesis and properties of a novel bolaamphiphile surfactant derived from proline. Chinese Chem. Lett..

[26] GEBICKI J.M., HICKS M. (1973). Ufasomes are Stable Particles surrounded by Unsaturated Fatty Acid Membranes. Nature.

[27] Prasad M., Hazra B., Tarafdar P.K. (2023). Molecular-level insights into a tripolyphosphate and pyrophosphate templated membrane assembly. Soft Matter.

[28] KASCHNY P., Goñi F.M. (1992). The components of merocyanine-540 absorption spectra in aqueous, micellar and bilayer environments. Eur. J. Biochem..

[29] Budin I., Prwyes N., Szostak J.W. (2014). Chain-Length Heterogeneity Allows for the Assembly of Fatty Acid Vesicles in Dilute Solutions. Biophys. J..

[30] Mansy S.S., Szostak J.W. (2008). Thermostability of model protocell membranes. Proc. Natl. Acad. Sci..

[31] Toparlak Ö.D., Karki M., Mansy S.S. (2020). Cyclophospholipids Increase Protocellular Stability to Metal Ions. Small.

[32] Lide D.R. (1995).

[33] Cistola D.P., Hamilton J.A., Small D.M. (1988). Ionization and phase behavior of fatty acids in water: application of the Gibbs phase rule. Biochemistry.

[34] Hamilton J.A. (1998). Fatty acid transport: difficult or easy?. J. Lipid Res..

[35] Hu J., Cochrane W.G., Paegel B.M. (2021). Chiral lipid bilayers are enantioselectively permeable. Nat. Chem..

[36] Henriques S.T., Peacock H., Craik D.J. (2019). Is the Mirror Image a True Reflection? Intrinsic Membrane Chirality Modulates Peptide Binding. J. Am. Chem. Soc..

[37] Mishima K., Tanaka S., Ogihara T. (2002). Electric field-induced orientation of L- and DL-phosphatidylcholine bilayers. Biochim. Biophys. Acta.

[38] Justice I., Kiesel P., Saenz J.P. (2024). A tuneable minimal cell membrane reveals that two lipid species suffice for life. Nat. Commun..

[39] Ma G., Allen H.C. (2006). DPPC Langmuir Monolayer at the Air−Water Interface: Probing the Tail and Head Groups by Vibrational Sum Frequency Generation Spectroscopy. Langmuir.

[40] Miranda P.B., Shen Y.R. (1999). Liquid Interfaces: A Study by Sum-Frequency Vibrational Spectroscopy. J. Phys. Chem. B.

[41] Meister A., Weygand M.J., Blume A. (2007). Evidence for a Reverse U-Shaped Conformation of Single-Chain Bolaamphiphiles at the Air−Water Interface. Langmuir.

[42] Hutter T., Linder C., Grinberg S. (2012). Interfacial and self-assembly properties of bolaamphiphilic compounds derived from a multifunctional oil. J. Colloid Interface Sci..

[43] Contini C., Pearson R., Battaglia G. (2018). Bottom-Up Evolution of Vesicles from Disks to High-Genus Polymersomes. iScience.

[44] Kanduč M., Stubenrauch C., Schneck E. (2024). Interface Adsorption versus Bulk Micellization of Surfactants: Insights from Molecular Simulations. J. Chem. Theory Comput..

[45] Toparlak O.D., Mansy S.S. (2019). Progress in synthesizing protocells. Exp. Biol. Med..

[46] Nader S., Baccouche A., Mansy S.S. (2023). Model Atmospheric Aerosols Convert to Vesicles upon Entry into Aqueous Solution. ACS Earth Sp. Chem..

[47] Babu D., Katsonis N., Ryabchun A. (2022). Motile behaviour of droplets in lipid systems. Nat. Rev. Chem.

