# Supplementary material for: A cationic single-chain bolalipid forms stable vesicles with distinct interfacial behavior
Source: Biophys J. 2026 Mar 26;125(9):2059–69. doi: 10.1016/j.bpj.2026.03.044 (PMC13351563; doi:10.1016/j.bpj.2026.03.044)
Supplement: Document S1. Figures S1–S14, Tables S1–S3, and supplemental methods [file mmc1.pdf]

Biophysical Journal, Volume *125*

Supplemental information

A cationic single-chain bolalipid forms stable vesicles with distinct interfacial behavior

Preeti Gahtori, Hossein Varghaei, Bibhas Hazra, Christian Jankovic, Jonathan Strobl, Harpreet Kaur, Sara Khamis, Frederick G. West, Julianne M. Gibbs, and Sheref S. Mansy

Figure S1. ^{1}H and ^{13}C NMR of 2Pro-C8:0

^{1}H NMR

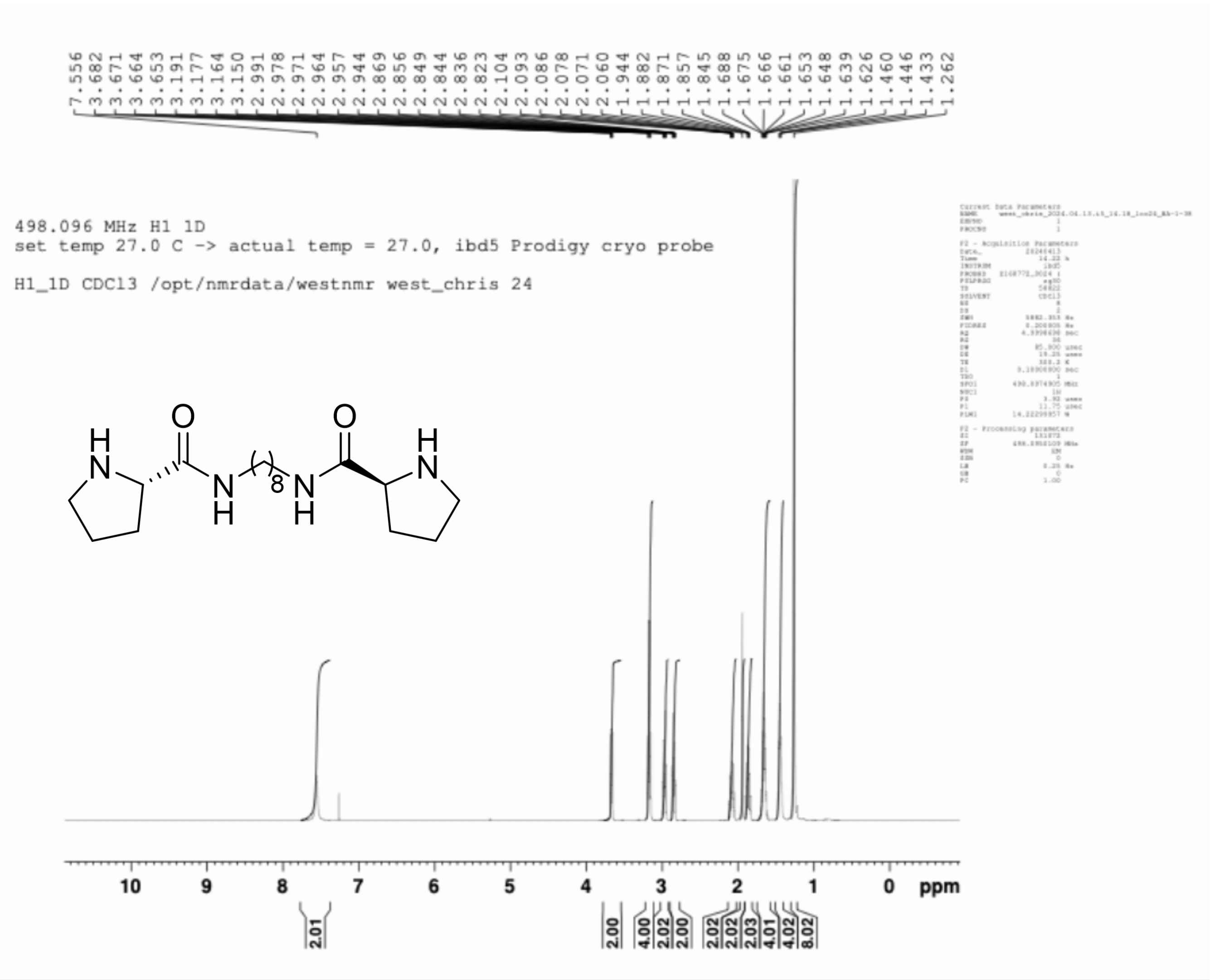

^{13}C NMR

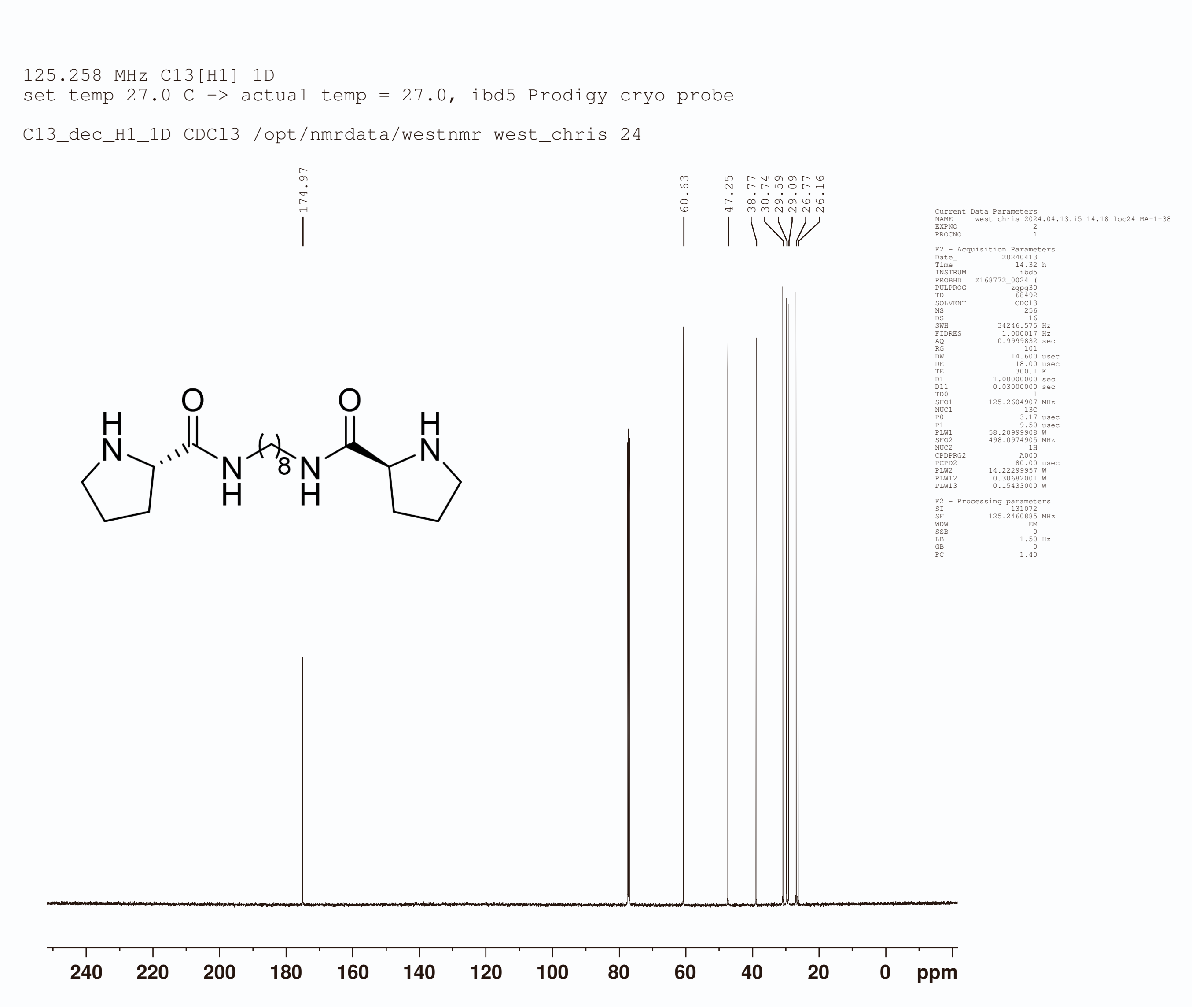

125.258 MHz C13[H1] 1D
set temp 27.0 C -> actual temp = 27.0, ibd5 Prodigy cryo probe
C13_dec_H1_1D CDCl3 /opt/nmrdata/westnmr west_chris 24
174.97
60.63
47.25
38.77
30.74
29.59
29.09
26.77
26.16
Current Data Parameters
NAME west_chris_2024.04.13.i5_14.18_loc24_BA-1-38
EXPNO 2
PROCNO 1
F2 - Acquisition Parameters
Date_ 20240413
Time 14.32 h
INSTRUM ibd5
PROBHD Z168772_0024 (
PULPROG zgpg30
TD 68492
SOLVENT CDCl3
NS 256
DS 16
SWH 34246.575 Hz
FIDRES 1.000017 Hz
AQ 0.9999832 sec
RG 101
DW 14.600 usec
DE 18.00 usec
TE 300.1 K
D1 1.00000000 sec
D11 0.03000000 sec
TD0 1
SFO1 125.2604907 MHz
NUC1 13C
P0 3.17 usec
P1 9.50 usec
PLW1 58.20999908 W
SFO2 498.0974905 MHz
NUC2 1H
CPDPRG2 A000
PCPD2 80.00 usec
PLW2 14.22299957 W
PLW12 0.30682001 W
PLW13 0.15433000 W
F2 - Processing parameters
SI 131072
SF 125.2460885 MHz
WDW EM
SSB 0
LB 1.50 Hz
GB 0
PC 1.40
240 220 200 180 160 140 120 100 80 60 40 20 0 ppm

Figure S2. ^{1}H and ^{13}C NMR of 2Pro-C10:0

^{1}H NMR

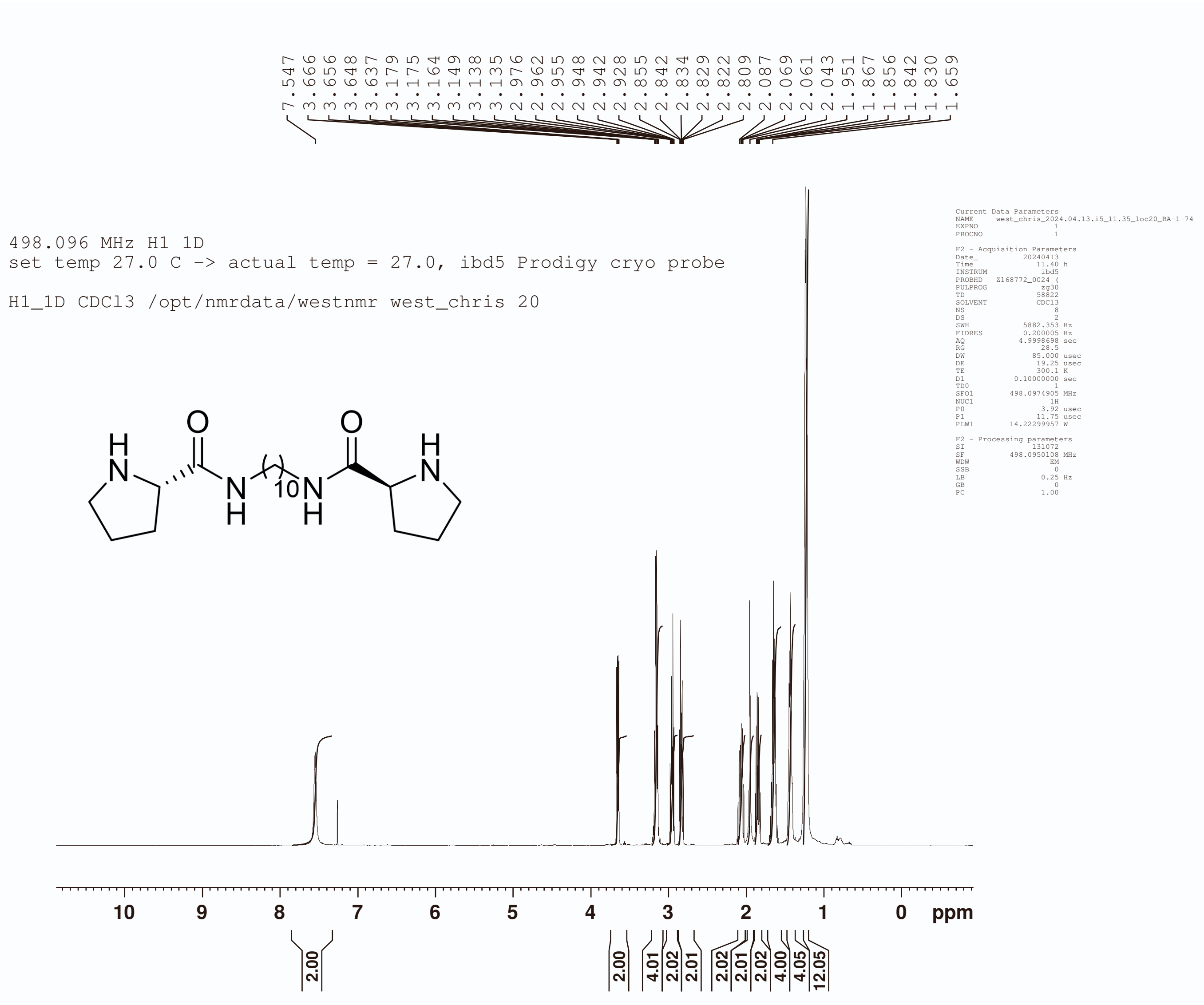

^{13}C NMR

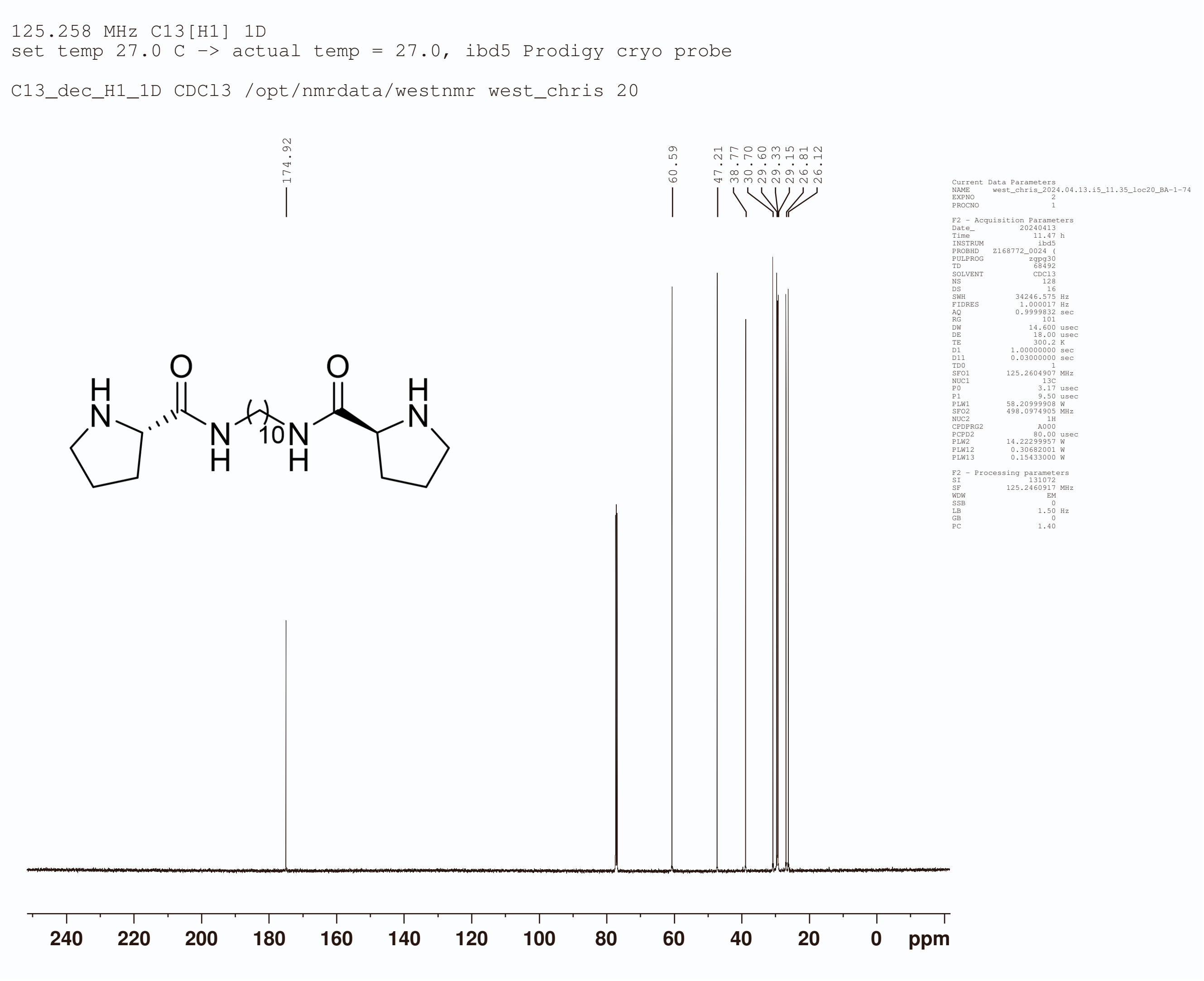
125.258 MHz C13[H1] 1D
set temp 27.0 C -> actual temp = 27.0, ibd5 Prodigy cryo probe
C13_dec_H1_1D CDCl3 /opt/nmrdata/westnmr west_chris 20
174.92
60.59
47.21
38.77
30.70
29.60
29.33
29.15
26.81
26.12
240 220 200 180 160 140 120 100 80 60 40 20 0 ppm

Figure S3. ^{1}H and ^{13}C NMR of 2Pro-C12:0

^{1}H NMR

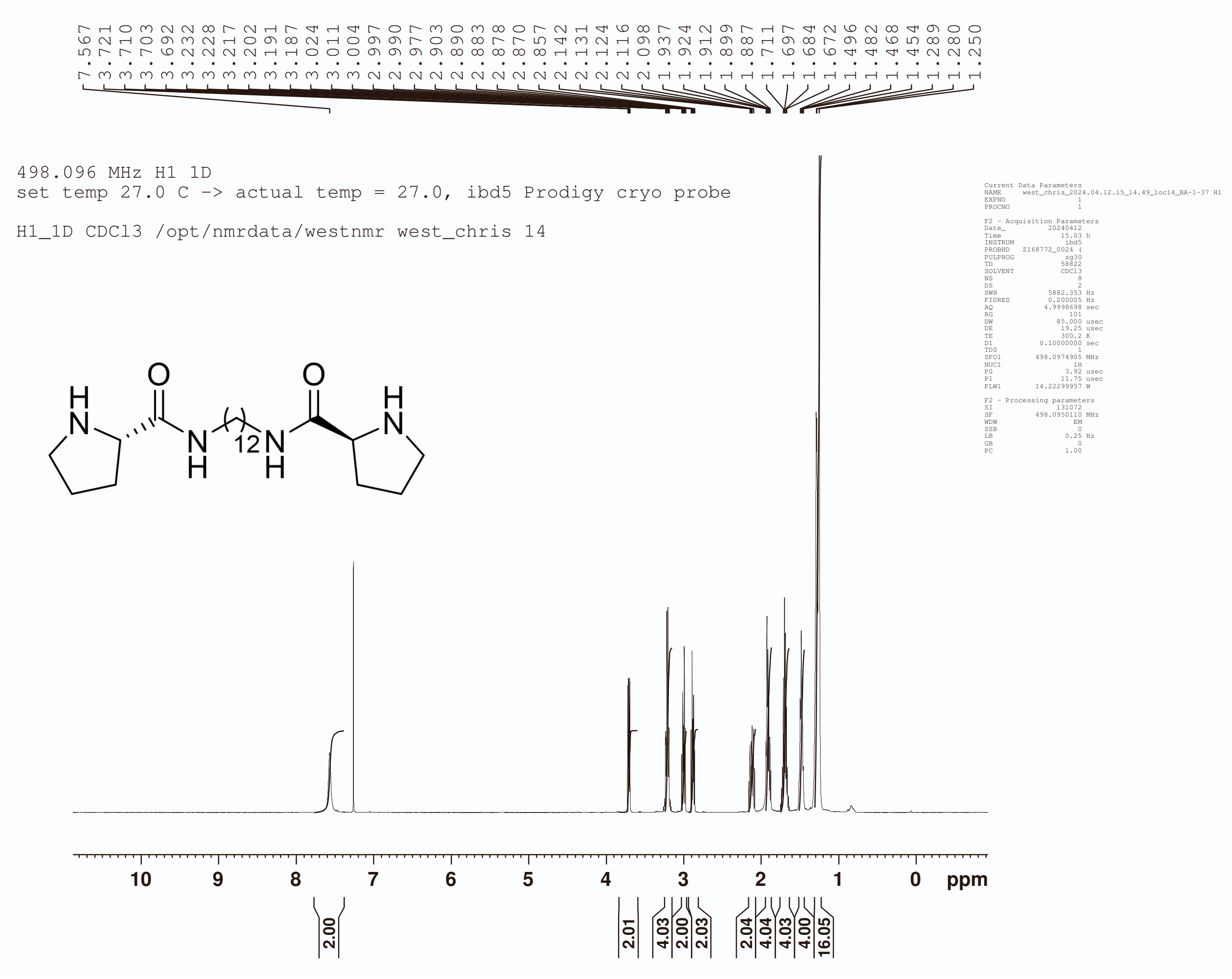

^{13}C NMR

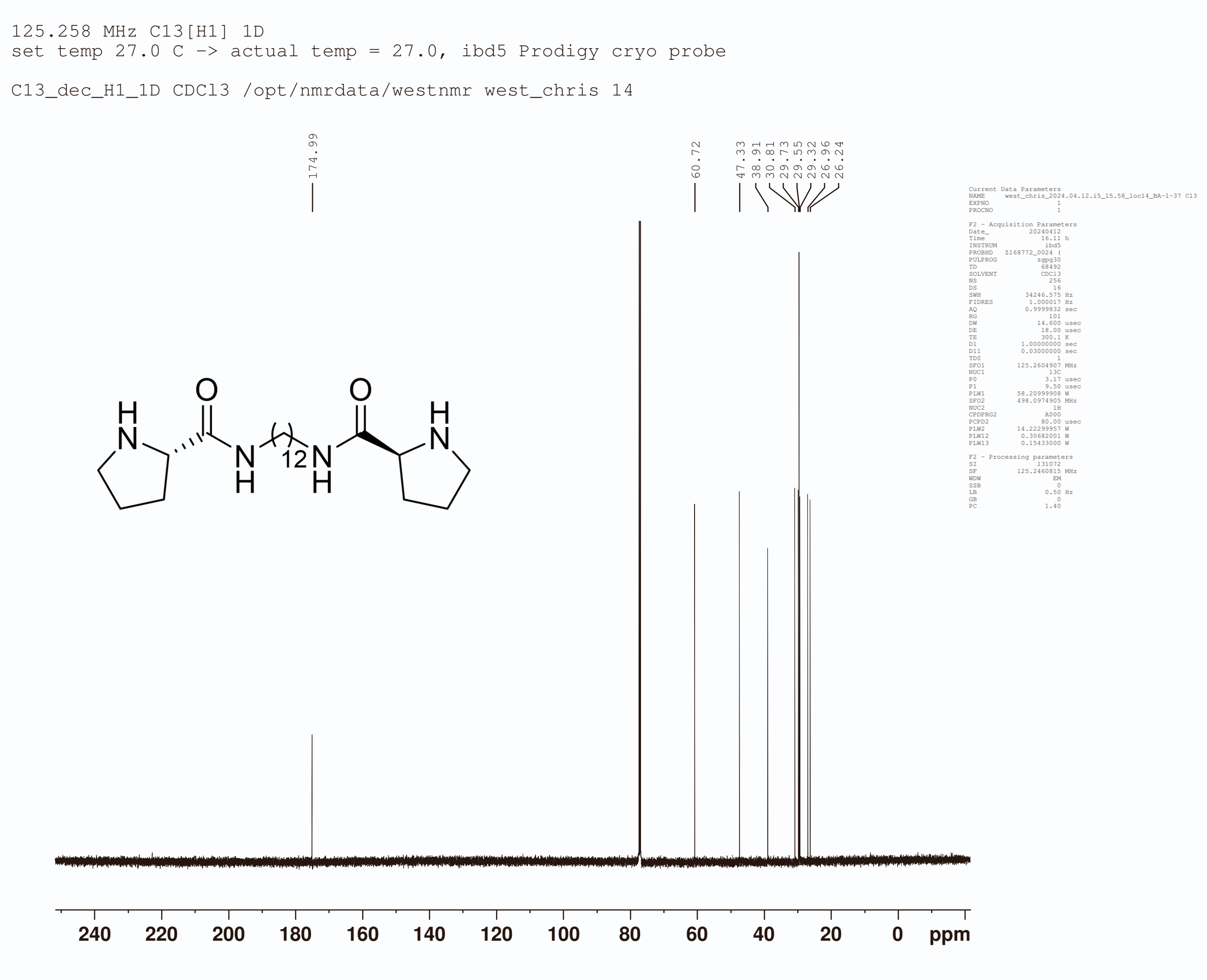
125.258 MHz C13[H1] 1D
set temp 27.0 C -> actual temp = 27.0, ibd5 Prodigy cryo probe
C13_dec_H1_1D CDCl3 /opt/nmrdata/westnmr west_chris 14
174.99
60.72
47.33
38.91
30.81
29.73
29.55
29.32
26.96
26.24
Current Data Parameters
NAME west_chris_2024.04.12.i5_15.58_loc14_BA-1-37 C13
EXPNO 1
PROCNO 1
F2 - Acquisition Parameters
Date_ 20240412
Time 16.11 h
INSTRUM ibd5
PROBHD Z168772_0024 (
PULPROG zgpg30
TD 68492
SOLVENT CDCl3
NS 256
DS 16
SWH 34246.575 Hz
FIDRES 1.000017 Hz
AQ 0.9999832 sec
RG 101
DW 14.600 usec
DE 18.00 usec
TE 300.1 K
D1 1.00000000 sec
D11 0.03000000 sec
TD0 1
SFO1 125.2604907 MHz
NUC1 13C
P0 3.17 usec
P1 9.50 usec
PLW1 58.20999908 W
SFO2 498.0974905 MHz
NUC2 1H
CPDPRG2 A000
PCPD2 80.00 usec
PLW2 14.22299957 W
PLW12 0.30682001 W
PLW13 0.15433000 W
F2 - Processing parameters
SI 131072
SF 125.2460815 MHz
WDW EM
SSB 0
LB 0.50 Hz
GB 0
PC 1.40
240 220 200 180 160 140 120 100 80 60 40 20 0 ppm

Figure S4. ^{1}H and ^{13}C NMR of 2Pro-C18:1

^{1}H NMR

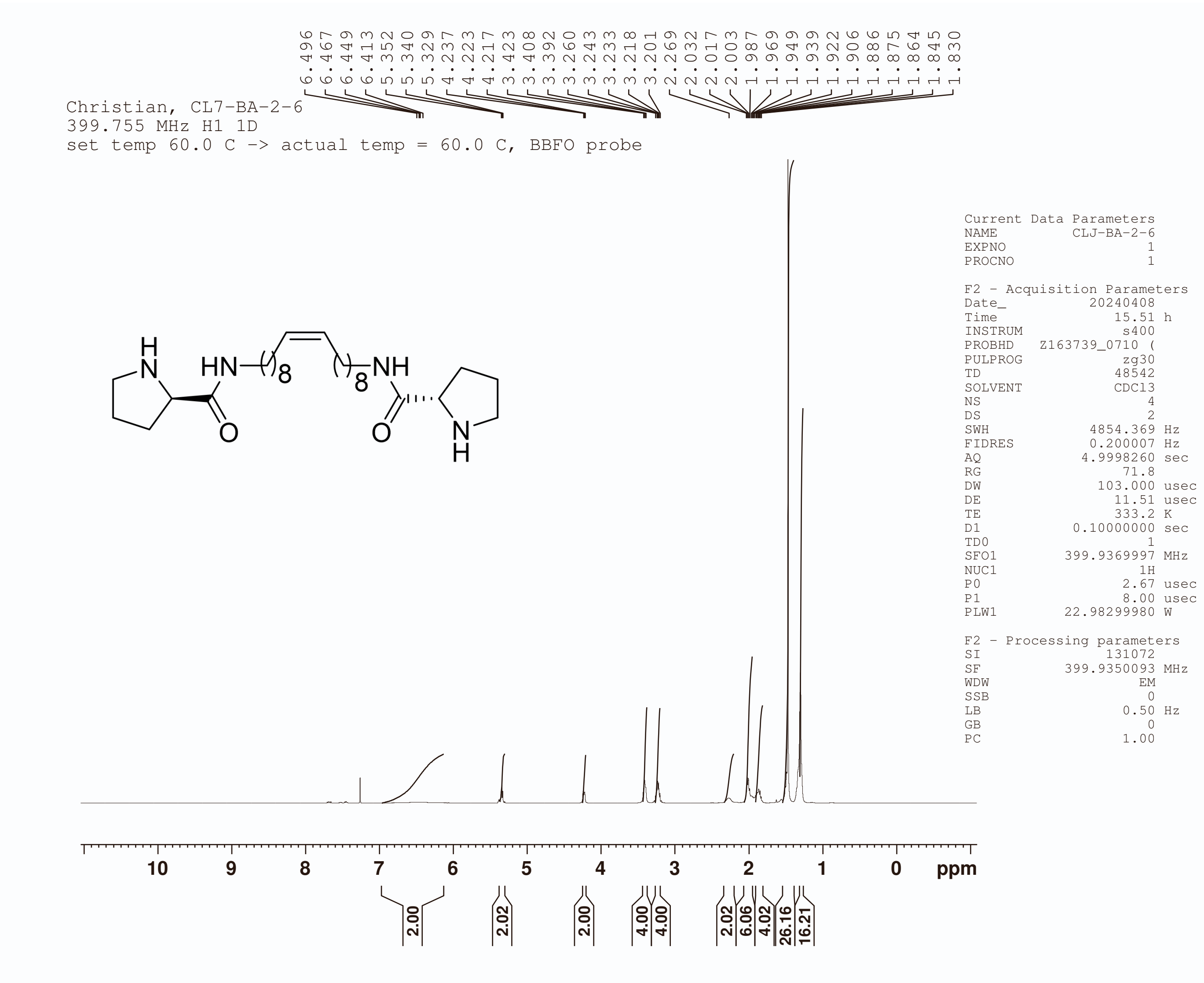

^{13}C NMR

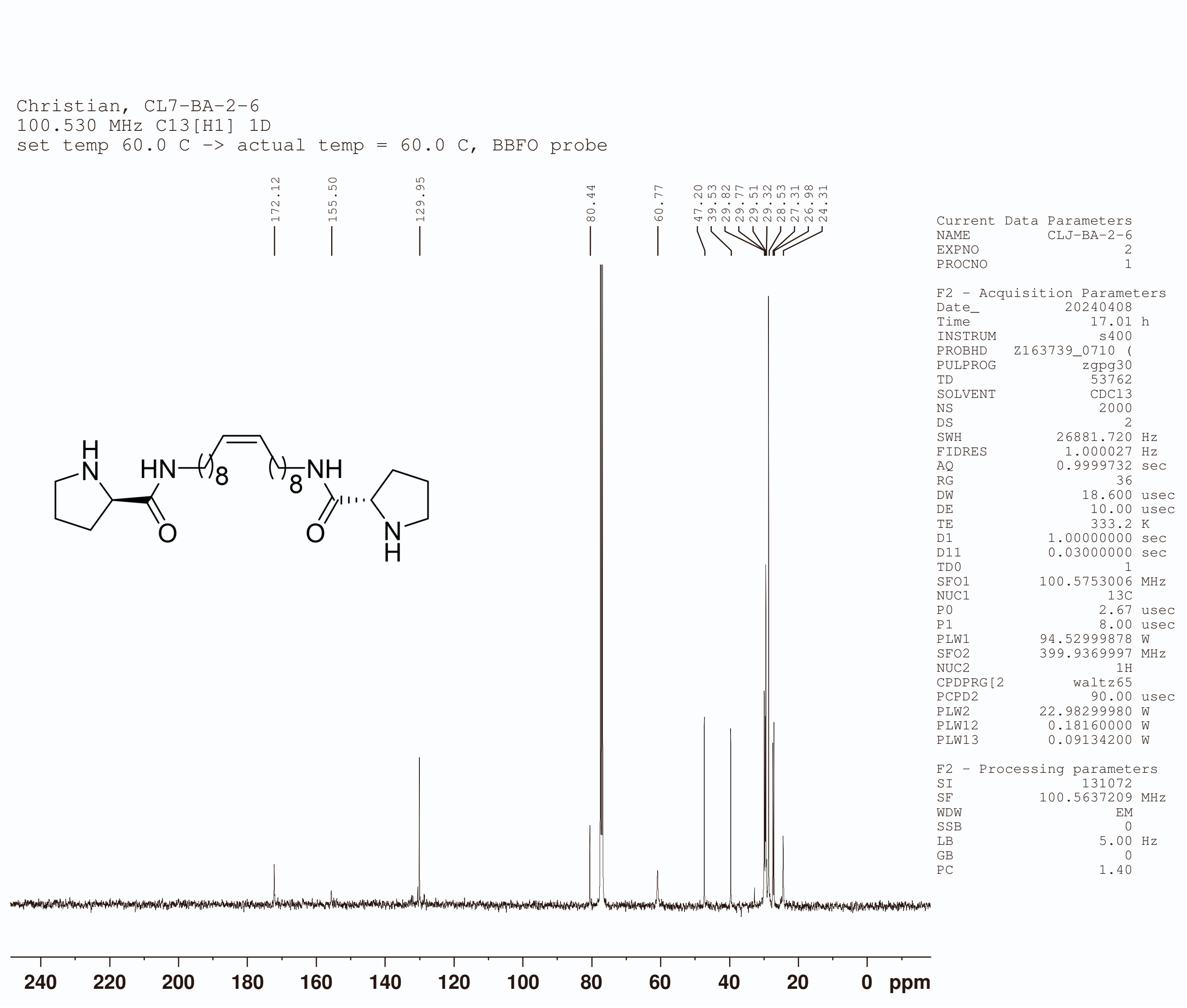

Christian, CL7-BA-2-6
100.530 MHz C13[H1] 1D
set temp 60.0 C -> actual temp = 60.0 C, BBFO probe
172.12
155.50
129.95
80.44
60.77
47.20
39.53
29.82
29.77
29.51
29.32
28.53
27.31
26.98
24.31
Current Data Parameters
NAME CLJ-BA-2-6
EXPNO 2
PROCNO 1
F2 - Acquisition Parameters
Date_ 20240408
Time 17.01 h
INSTRUM s400
PROBHD Z163739_0710 (
PULPROG zgpg30
TD 53762
SOLVENT CDCl3
NS 2000
DS 2
SWH 26881.720 Hz
FIDRES 1.000027 Hz
AQ 0.9999732 sec
RG 36
DW 18.600 usec
DE 10.00 usec
TE 333.2 K
D1 1.00000000 sec
D11 0.03000000 sec
TD0 1
SFO1 100.5753006 MHz
NUC1 13C
P0 2.67 usec
P1 8.00 usec
PLW1 94.52999878 W
SFO2 399.9369997 MHz
NUC2 1H
CPDPRG[2 waltz65
PCPD2 90.00 usec
PLW2 22.98299980 W
PLW12 0.18160000 W
PLW13 0.09134200 W
F2 - Processing parameters
SI 131072
SF 100.5637209 MHz
WDW EM
SSB 0
LB 5.00 Hz
GB 0
PC 1.40
240 220 200 180 160 140 120 100 80 60 40 20 0 ppm

Figure S5. ^{1}H and ^{13}C NMR of 1Pro-C18:1

^{1}H NMR

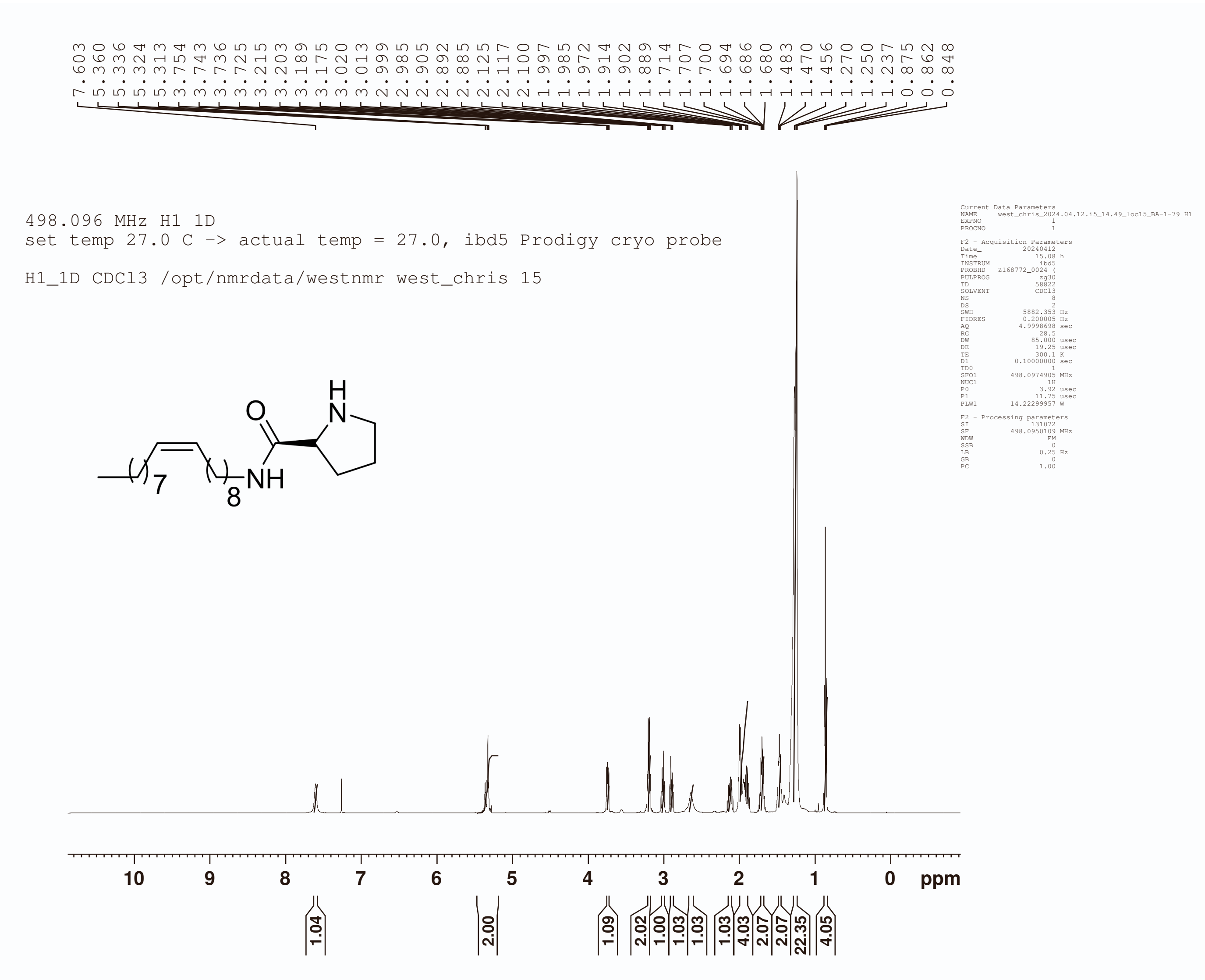

^{13}C NMR

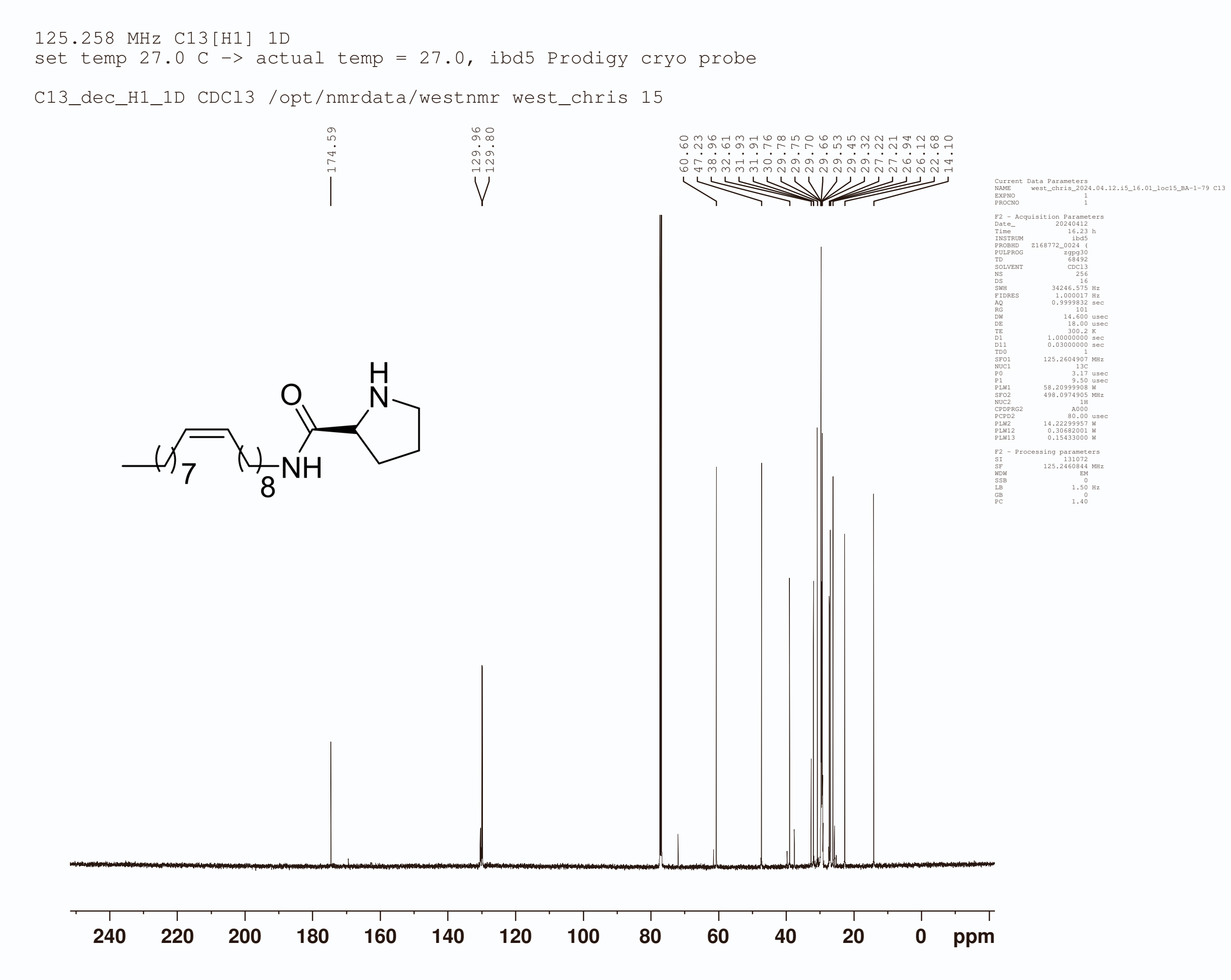
125.258 MHz C13[H1] 1D
set temp 27.0 C -> actual temp = 27.0, ibd5 Prodigy cryo probe
C13_dec_H1_1D CDCl3 /opt/nmrdata/westnmr west_chris 15
174.59
129.96
129.80
60.60
47.23
38.96
32.61
31.93
31.91
30.76
29.78
29.75
29.70
29.66
29.53
29.45
29.32
27.22
27.21
26.94
26.12
22.68
14.10
Current Data Parameters
NAME west_chris_2024.04.12.i5_16.01_loc15_BA-1-79 C13
EXPNO 1
PROCNO 1
F2 - Acquisition Parameters
Date_ 20240412
Time 16.23 h
INSTRUM ibd5
PROBHD Z168772_0024 (
PULPROG zgpg30
TD 68492
SOLVENT CDCl3
NS 256
DS 16
SWH 34246.575 Hz
FIDRES 1.000017 Hz
AQ 0.9999832 sec
RG 101
DW 14.600 usec
DE 18.00 usec
TE 300.2 K
D1 1.00000000 sec
D11 0.03000000 sec
TD0 1
SFO1 125.2604907 MHz
NUC1 13C
P0 3.17 usec
P1 9.50 usec
PLW1 58.20999908 W
SFO2 498.0974905 MHz
NUC2 1H
CPDPRG2 A000
PCPD2 80.00 usec
PLW2 14.22299957 W
PLW12 0.30682001 W
PLW13 0.15433000 W
F2 - Processing parameters
SI 131072
SF 125.2460844 MHz
WDW EM
SSB 0
LB 1.50 Hz
GB 0
PC 1.40
O
H
N
7
8
NH
240
220
200
180
160
140
120
100
80
60
40
20
0
ppm

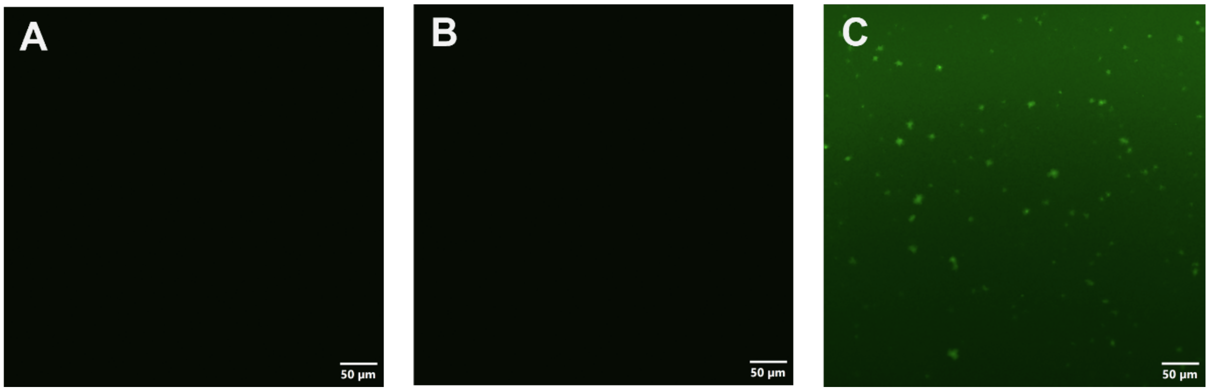

Figure S6. Confocal microscopy images of 2Pro-C8:0 (A), 2Pro-C10:0 (B) and 2Pro-C12:0 (C) at pH 8. The scale bar represents 5 μm in all the images.

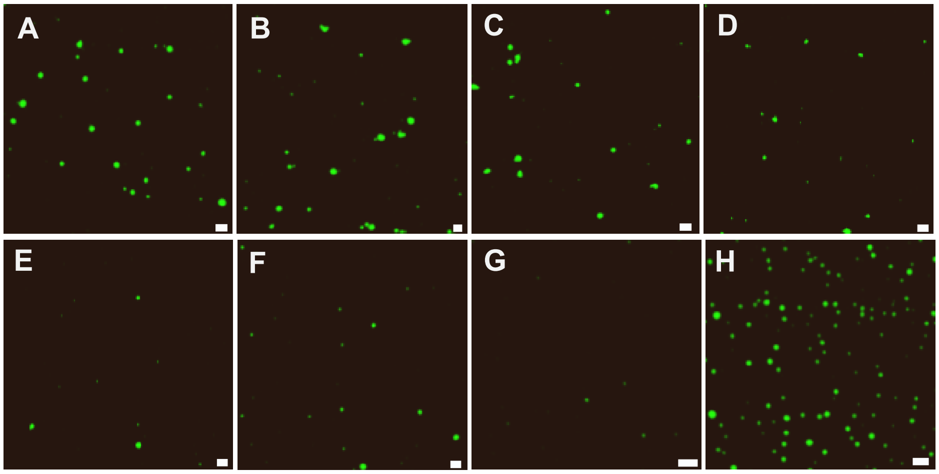

Figure S7. Confocal microscopy images of 1Pro-C18:1 (A-D) and 2Pro-C18:1 (E-H) in the presence of (A, E) 250 mM NaCl, (B, F) 250 mM KCl, (C, G) 10 mM Mg^{2+}, (D, H) 10 mM Ca^{2+} at pH 7.5. Samples were diluted two-fold prior to imaging. The scale bar represents 5 μm in all the images.

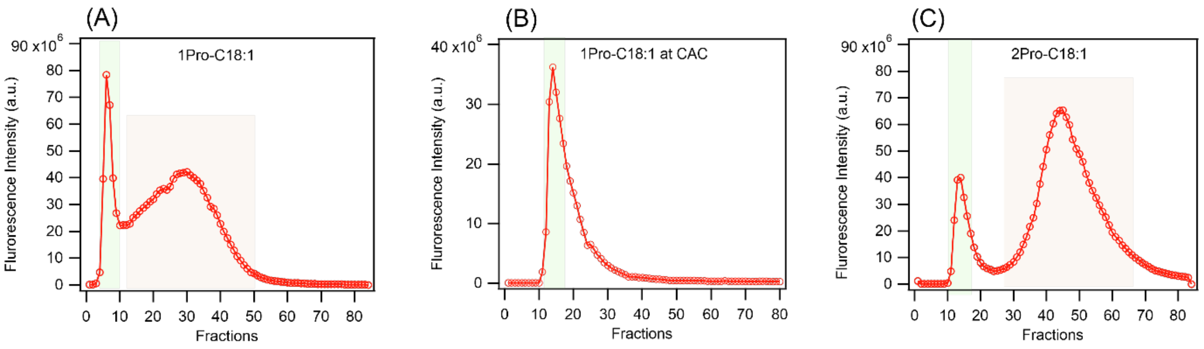

Figure S8. Size exclusion chromatograms of HPTS-loaded vesicles formed by (A) 1Pro-C18:1 and (B) 1Pro-C18:1 above the critical aggregation concentration (CAC) in running buffer (C) 2Pro-C18:1 at pH 8.0 showing distinct peaks corresponding to vesicle-entrapped and free fluorophore.

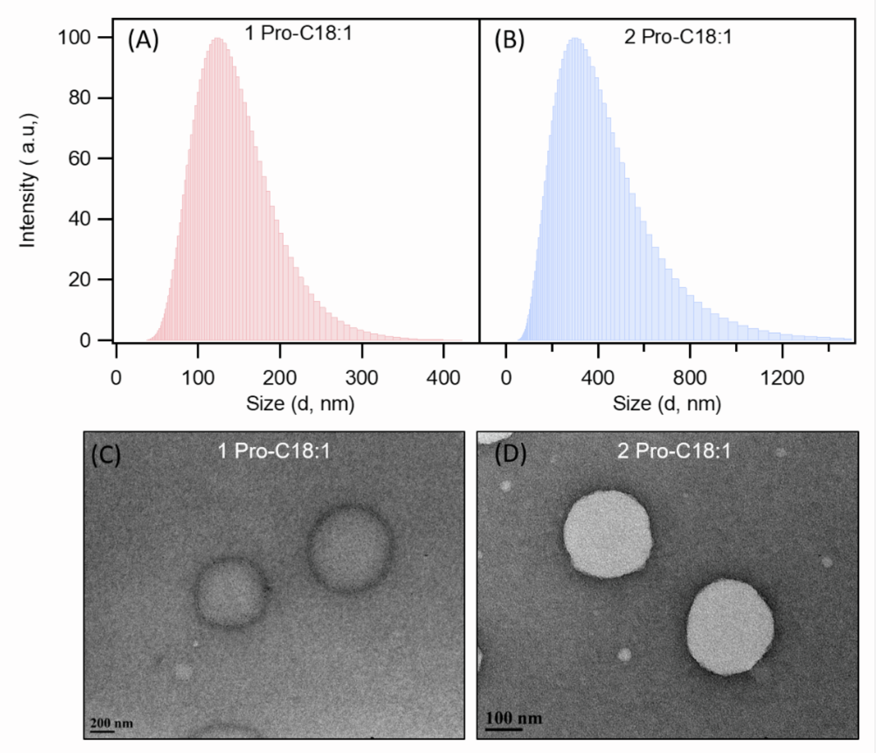

Figure S9. Structural characterization of vesicles formed by 1Pro-C18:1 and 2Pro-C18:1 in aqueous solution. Intensity-weighted dynamic light scattering (DLS) size distributions of aggregates formed by (A) 1Pro-C18:1 and (B) 2Pro-C18:1 under vesicle-forming conditions. Transmission electron microscopy (TEM) images of vesicles formed by (C) 1Pro-C18:1 and (D) 2Pro-C18:1 lipids.

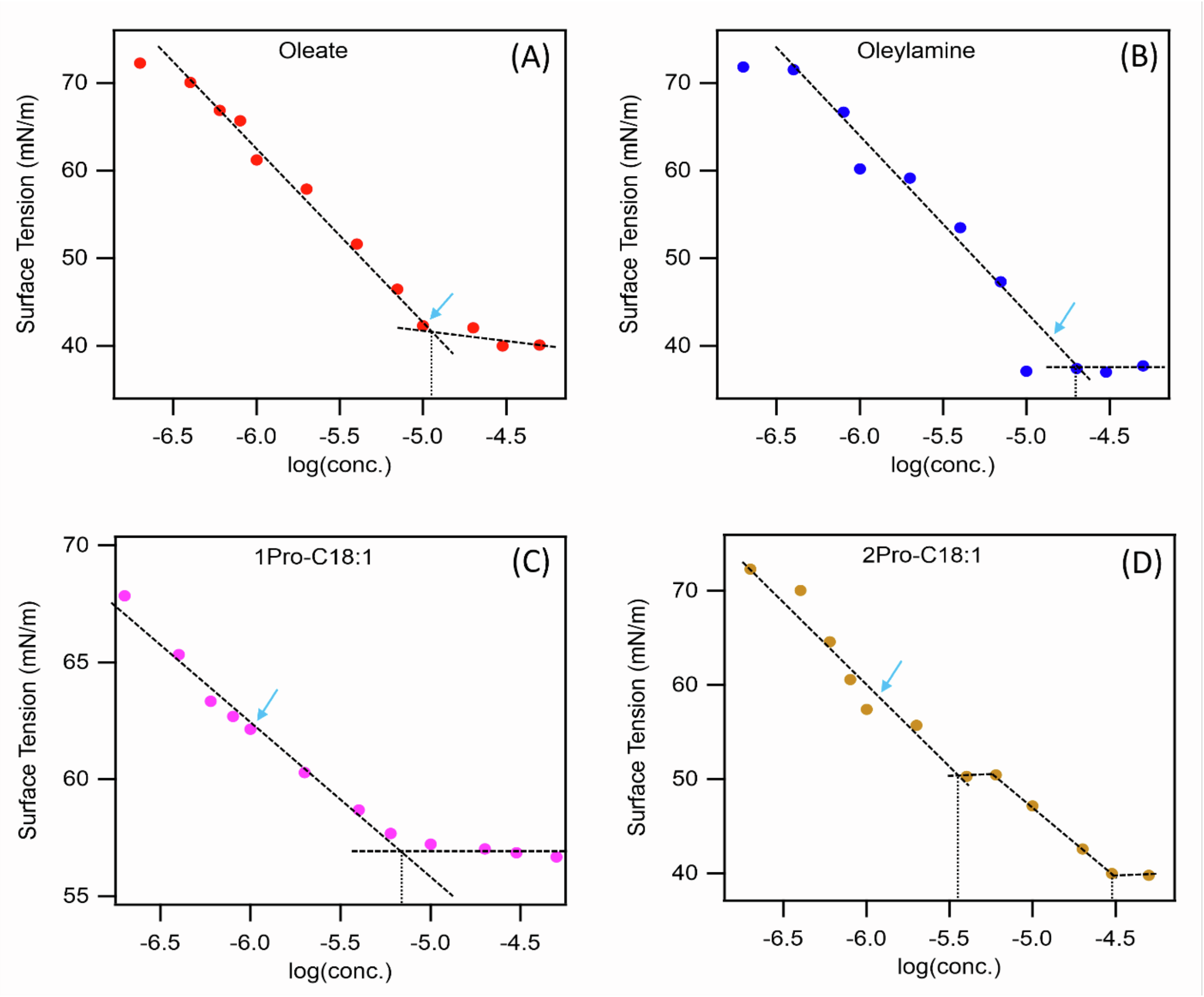

Figure S10. Surface tension measurements at the air–water interface at pH 8.0 for (A) oleic acid, (B) oleylamine, (C) 1Pro-C18:1, and (D) 2Pro-C18:1 before and after monolayer saturation. The blue arrows indicate the monolayer saturation concentration in each case. Inflection points in the surface tension isotherms are indicated by vertical lines.

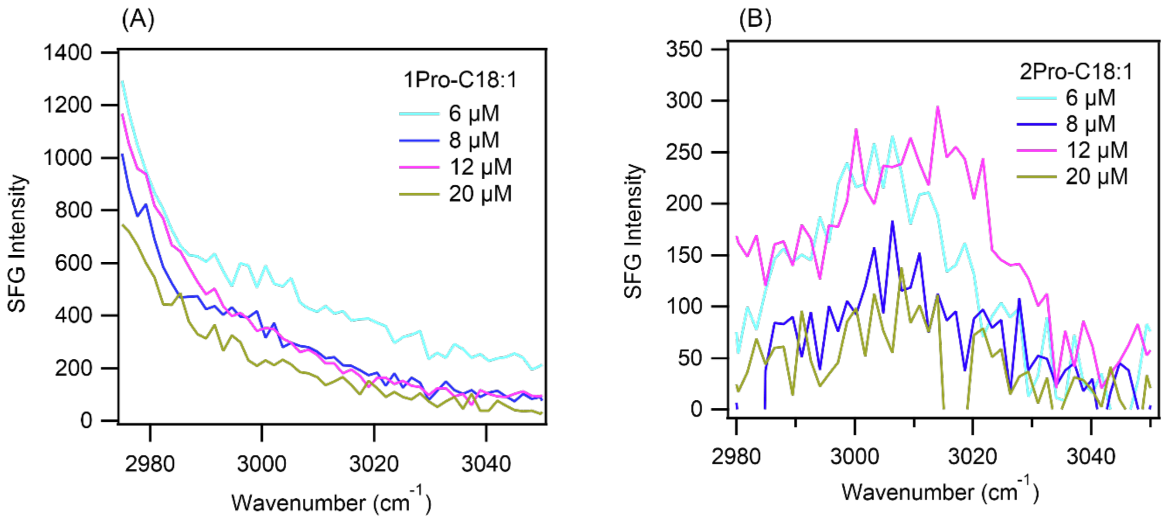

Figure S11. vSFG spectra in the =C–H stretching region for (A) 1Pro-C18:1 and (B) 2Pro-C18:1 at the air–water interface. The characteristic peak at ~3010 cm^{-1}, corresponding to the alkene C–H stretch, is absent in 1Pro-C18:1 and indicates a bent ('horseshoe') molecular configuration at the interface for 2Pro-C18:1.

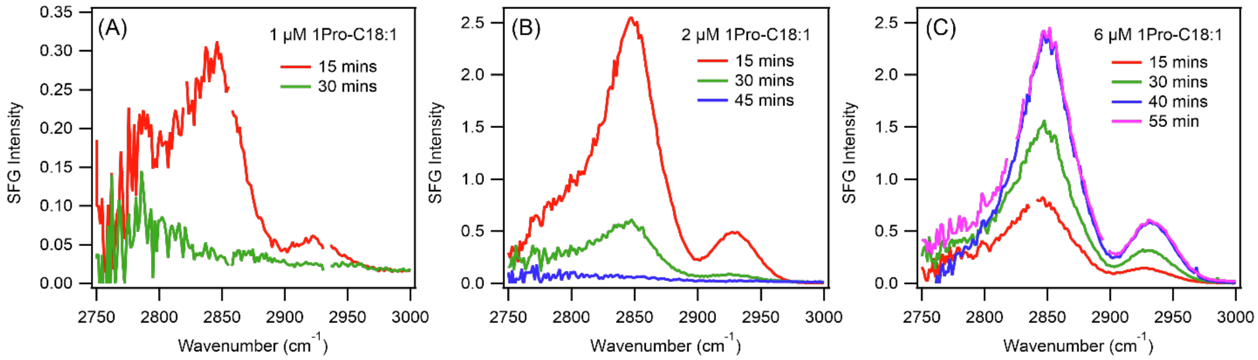

Figure S12. vSFG spectra in the C–H stretching region for 1Pro-C18:1 at the air–water interface at concentrations of (A) 1 μM, (B) 2 μM, and (C) 6 μM. Spectra were collected at selected time intervals after lipid injection. At lower concentrations (1–2 μM), the initial C–H signals disappear over time, indicating rapid molecular reorganization or desorption. At 6 μM, well-defined C–H features stabilize within ~55 minutes, suggesting adsorption of monomer at interface.

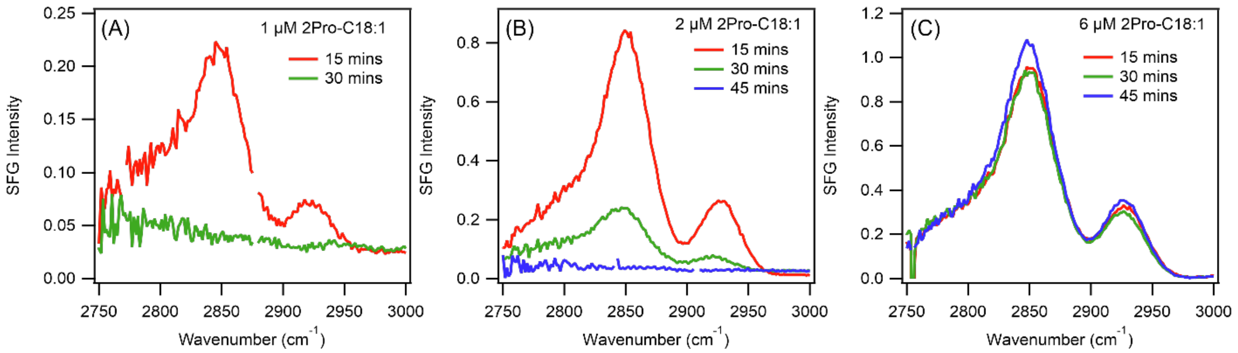

Figure S13. vSFG spectra in the C–H stretching region for 2Pro-C18:1 at the air–water interface at concentrations of (A) 1 μM, (B) 2 μM, and (C) 6 μM. Spectra were collected at selected time intervals after lipid injection. At lower concentrations (1–2 μM), the initial C–H signals disappear over time, indicating rapid molecular reorganization or desorption. At 6 μM, well-defined C–H features stabilize within ~45 minutes, suggesting adsorption of monomer at interface.

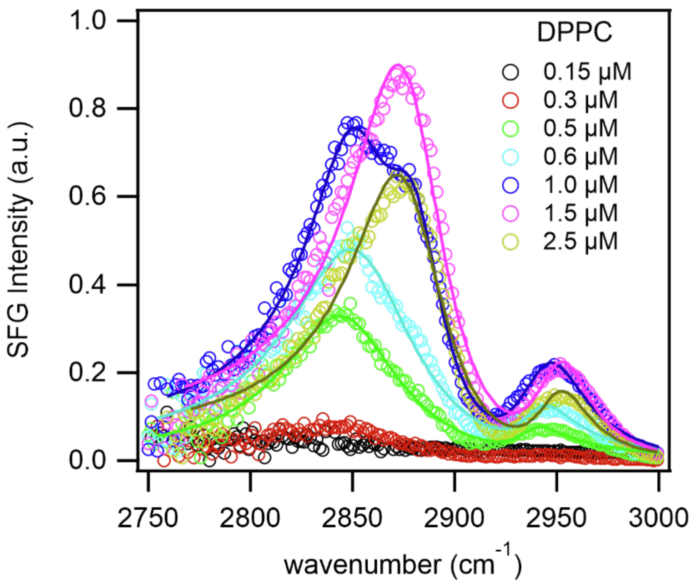

Figure S14. vSFG spectra in the C–H stretching region (2800–3000 cm⁻¹) of DPPC lipid monolayers at the air–water interface as a function of lipid concentration. The increasing C–H vibrational signal with concentration reflects enhanced lipid adsorption and molecular ordering at the interface.

Supplementary Tables

Table S1. Signals frequencies (ω) and their corresponding molecular amplitudes (A) of the vSFG spectra of the **1Pro-C18:1 lipid**. The non-resonant amplitude (χ_{NR}) was found to be -0.1.

Conc. of 1Pro-C18:1 lipid (μM)	Signals frequencies (ω) (cm^{-1})		Molecular amplitudes (A) (a.u.)		$A_{CH_2^{as}} / A_{CH_2^{ss}}$
	CH_2^{ss}	CH_2^{as}	CH_2^{ss}	CH_2^{as}	
1	-	-	-	-	
2	-	-	-	-	
4	2852.9 ± 0.553	2926.8 ± 1.57	21.209 ± 0.217	5.1517 ± 0.23	0.24057
6	2852.7 ± 0.722	2928.8 ± 1.45	39.105 ± 0.423	10.825 ± 0.506	0.27621
8	2852.7 ± 0.219	2926.4 ± 0.908	36.322 ± 0.127	8.2563 ± 0.24	0.2259
12	2853 ± 0.402	2926.6 ± 0.914	38.328 ± 0.194	8.7262 ± 0.299	0.22715
20	2851.4 ± 0.5	2925.7 ± 1.24	31.257 ± 0.187	6.4987 ± 0.312	0.17949

Table S2. Signals frequencies (ω) and their corresponding molecular amplitudes (A) of the vSFG spectra of the **2Pro-C18:1 lipid**. The non-resonant amplitude (χ_{NR}) was found to be -0.1.

Conc. of 2Pro-C18:1 lipid (μM)	Signals frequencies (ω) (cm^{-1})		Molecular amplitudes (A) (a.u.)		$A_{CH_2^{as}} / A_{CH_2^{ss}}$
	CH_2^{ss}	CH_2^{as}	CH_2^{ss}	CH_2^{as}	
1	-	-	-	-	
2	-	-	-	-	
4	2857.8 ± 0.266	2925.4 ± 0.409	21.802 ± 0.359	8.1288 ± 0.304	0.37156
6	2856.2 ± 0.321	2923.6 ± 0.461	24.438 ± 0.442	8.4103 ± 0.381	0.34426
8	2858.3 ± 0.322	2925.6 ± 0.54	18.662 ± 0.423	7.3291 ± 0.35	0.39247
12	2858 ± 0.336	2925.9 ± 0.454	23.947 ± 0.446	8.4999 ± 0.387	0.35565
20	2857.2 ± 0.428	2924.4 ± 0.623	15.778 ± 0.388	5.2289 ± 0.329	0.33121

Table S3. Signals frequencies (ω) (top) and their corresponding molecular amplitudes (A) (bottom) of the vSFG spectra of the **DPPC lipid**. The non-resonant amplitude (χ_{NR}) was found to be -0.1.

**Conc. of DPPC lipid (μM)**	**Signals frequencies (ω) (cm^{-1})**		
	**CH_2^{ss}**	**CH_3^{ss}**	**CH_3^{FR}**
0.15	-	-	-
0.3	-	-	-
0.5	2850.2 ± 1.36	-	2943.7 ± 2.08
0.6	2853.6 ± 1.29	-	2941.3 ± 1.64
1.0	2853.9 ± 0.766	2880.6 ± 0.519	2944.8 ± 0.833
1.5	-	2878.4 ± 0.561	2951.4 ± 0.983
2.5	-	2877.6 ± 0.663	2950.8 ± 1.13

**Conc. of DPPC lipid (μM)**	**Molecular amplitudes (A) (a.u.)**		
	**CH_2^{ss}**	**CH_3^{ss}**	**CH_3^{FR}**
0.15	-	-	-
0.3	-	-	-
0.5	20.05 ± 0.458	-	1.9876 ± 0.494
0.6	25.568 ± 0.639	-	2.9133 ± 0.586
1.0	14.131 ± 2.01	9.3191 ± 1.45	7.4108 ± 0.488
1.5	-	24.362 ± 0.646	5.8172 ± 0.555
2.5	-	21.241 ± 0.59	4.4863 ± 0.525

Supplemental methods

Synthesis and Characterization of Lipids

General Procedure for EDC Couplings to Synthesize SI-1 to SI-3

Corresponding amine (1.0 equiv), Boc-L-proline (2.2 equiv), DMAP (0.1 equiv), and DIPEA (2.5 equiv) were dissolved in CH_2Cl_2 (0.1 M with respect to amine). EDC•HCl (2.2 equiv) was added to the reaction mixture and stirred at room temperature(rt). After 24 h, 1 M HCl was added to the reaction mixture, the layers separated, and the aqueous layer was extracted with CH_2Cl_2 x 3. The combined organic layers were washed with brine x 1 and dried over Na_2SO_4. Filtration and evaporation of the solvent gave a crude mixture which was purified by silica gel column chromatography to provide the corresponding amide.

SI-1

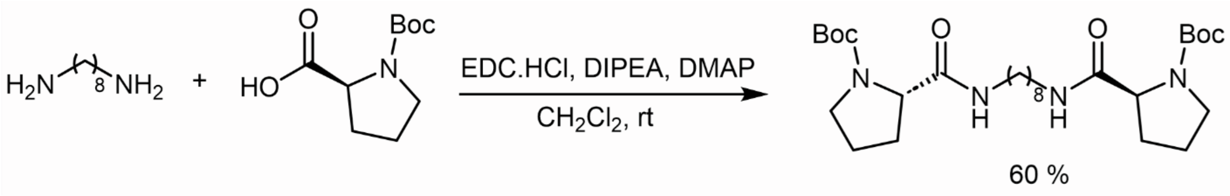

Using 1,8-diaminooctane (0.533 g, 3.70 mmol), Boc-L-proline (1.75 g, 8.13 mmol), DMAP (0.0450 g, 0.370 mmol), DIPEA (1.61 mL, 9.24 mmol), and EDC•HCl (1.56 g, 8.13 mmol) furnished **SI-1** (1.19 g, 60% yield) as a yellow oil after column chromatography employing 9:1 EtOAc/MeOH as the eluent.

R_f = 0.48 (9:1 EtOAc/MeOH); ^{1}H NMR (400 MHz, $CDCl_3$, 333 K) δ 6.95–5.92 (br s, 2H), 4.26–4.18 (m, 2H), 3.43–3.36 (m, 4H), 3.26–3.17 (m, 4H), 2.32–2.18 (m, 2H), 2.07–1.78 (m, 6H), 1.53–1.39 (m, 24H), 1.34–1.24 (m, 6H); ^{13}C NMR (100 MHz, $CDCl_3$, 333 K) δ 172.2, 155.5, 80.4, 60.7, 47.2, 39.5, 29.7, 29.2, 28.5, 26.9, 24.3; HRMS (ESI) calcd for $C_{28}H_{50}N_4NaO_6$ $[M + Na]^+$ 561.3623, found 561.3626.

SI-2

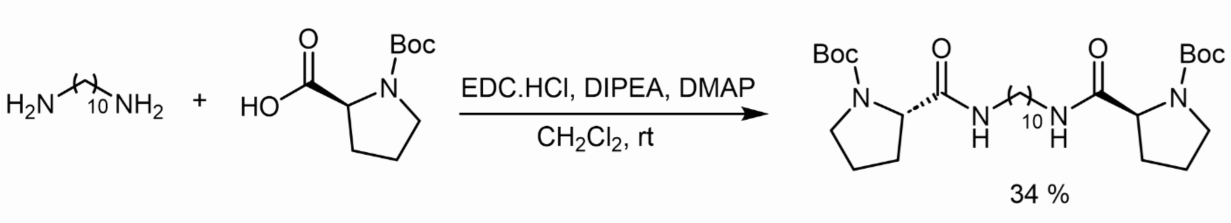

Using 1,10-diaminodecane (1.21 g, 7.02 mmol), Boc-L-proline (3.28 g, 15.5 mmol), DMAP (0.0858 g, 0.702 mmol), DIPEA (3.06 mL, 17.6 mmol), and EDC•HCl (2.96 g, 15.5 mmol) furnished **SI-2** (1.36 g, 34% yield) as a yellow oil after column chromatography employing 9:1 EtOAc/MeOH as the eluent.

R_f = 0.53 (9:1 EtOAc/MeOH); ^{1}H NMR (400 MHz, $CDCl_3$, 333 K) δ 6.64–6.26 (m, 2H), 4.26–4.17 (m, 2H), 3.42–3.36 (m, 4H), 3.25–3.17 (m, 4H), 2.29–2.18 (m, 2H), 1.97–1.79 (m, 4H), 1.51–1.40 (m, 24H), 1.32–1.23 (m, 12H); ^{13}C NMR (100 MHz, $CDCl_3$, 333 K) δ 172.1, 155.4, 80.4, 60.8, 41.2, 39.5, 29.7, 29.5, 29,3m 28.5, 26.9, 24.3; HRMS (ESI) calcd for $C_{30}H_{54}N_4NaO_6$ $[M + Na]^+$ 589.3936, found 589.3934.

SI-3

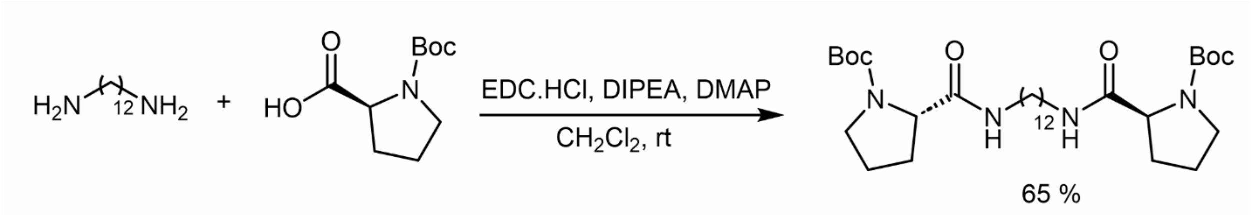

Using 1,12-diaminododecane (0.327 g, 1.63 mmol), Boc-L-proline (0.772 g, 3.59 mmol), DMAP (0.0199 g, 0.163 mmol), DIPEA (0.710 mL, 4.08 mmol), and EDC•HCl (0.687 g, 3.59 mmol) furnished **SI-3** (0.633 g, 65% yield) as a colorless oil after column chromatography employing 9:1 EtOAc/MeOH as the eluent.

R_f = 0.60 (9:1 EtOAc/MeOH); ^{1}H NMR (400 MHz, $CDCl_3$, 333 K) δ 6.64–6.24 (m, 2H), 4.27–4.18 (m, 2H), 3.45–3.35 (m, 4H), 3.26–3.18 (m, 4H), 2.32–2.21 (m, 2H), 1.93–1.81 (m, 6H), 1.53–1.42 (m, 24H), 1.32–1.23 (m, 14H); ^{13}C NMR (100 MHz, $CDCl_3$, 333 K) δ 172.1, 155.5, 80.4, 60.8, 47.2,

39.5, 29.7, 29.58, 29.56, 29.3, 27.0, 24.3; HRMS (ESI) calcd for $C_{32}H_{58}N_4NaO_6$ $[M + Na]^+$ 617.4249, found 617.4248.

General Procedure for Boc Deprotection of SI-1 to SI-3

The corresponding amide was dissolved in a 1:1 mixture of CH_2Cl_2: TFA (0.1 M) and stirred at rt for 24 h, after which the resulting mixture was concentrated under reduced pressure. The crude mixture was then dissolved in CH_2Cl_2 and washed with 1 M $NaOH_{(aq)}$. The aqueous layer was extracted with CH_2Cl_2 x 3, the combined organic extracts washed with brine x 1, then dried over Na_2SO_4. Filtration and evaporation of the solvent gave the corresponding bolaamphiphile as a pure product.

2Pro-C8:0

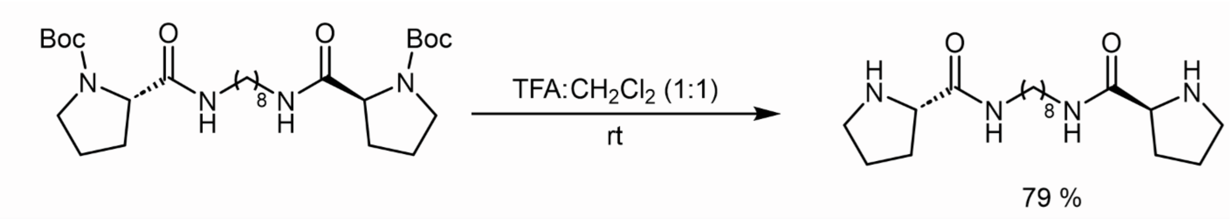

Using **SI-1** (1.19 g, 2.21 mmol) furnished **2Pro-C8:0 (**0.588 g, 79%) as an orange solid after workup.

R_f = 0.12 (9:1 EtOAc/MeOH); ^{1}H NMR (500 MHz, $CDCl_3$) δ 7.59–7.51 (m, 2H), 3.67 (dd, J = 9.4, 5.2 Hz, 2H), 3.20–3.15 (m, 4H), 3.0–2.94 (m, 2H), 2.88–2.81 (m, 2H), 2.12–2.04 (m, 2H), 1.96–1.93 (m, 2H), 1.90–1.83 (m, 2H), 1.71–1.61 (m, 4H), 1.49–1.40 (m, 4H), 1.30–1.20 (m, 8H); ^{13}C NMR (125 MHz, $CDCl_3$) δ 175.0, 60.6, 47.2, 38.8, 30.7, 29.6, 29.1, 26.8, 26.2; HRMS (ESI) calcd for $C_{18}H_{35}N_4O_2$ $[M + H]^+$ 339.2760, found 339.2758.

2Pro-C10:0

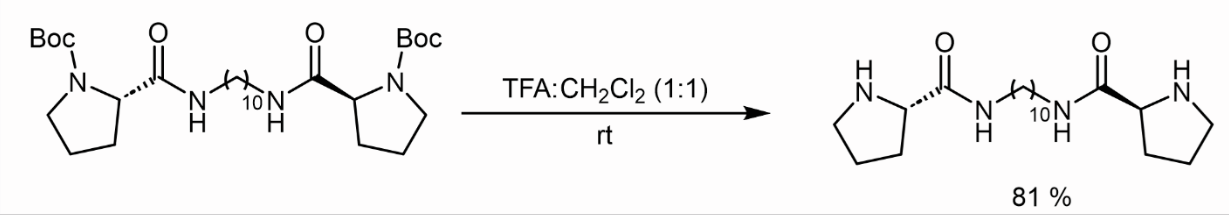

Using **SI-2** (1.36 g, 2.40 mmol) furnished **2Pro-C8:0** (0.709 g, 81%) as a white solid after workup.

R_f = 0.19 (9:1 EtOAc/MeOH); ^{1}H NMR (500 MHz, $CDCl_3$) δ 7.59–7.49 (m, 2H), 3.65 (dd, J = 9.2, 5.3 Hz, 2H), 3.19–3.12 (m, 4H), 2.99–2.92 (m, 2H), 2.86–2.80 (m, 2H), 2.11–2.02 (m, 2H), 1.98–1.92 (m, 2H), 1.89–1.80 (m, 2H), 1.70–1.59 (m, 4H), 1.47–1.39 (m, 4H), 1.28–1.17 (m, 12H); ^{13}C NMR (125 MHz, $CDCl_3$) δ 174.9, 60.6, 47.2, 38.8, 30.7, 29.6, 29.3, 29.1, 26.8, 26.1; HRMS (ESI) calcd for $C_{20}H_{39}N_4O_2$ $[M + H]^+$ 367.3068, found 367.3067.

2Pro-C12:0

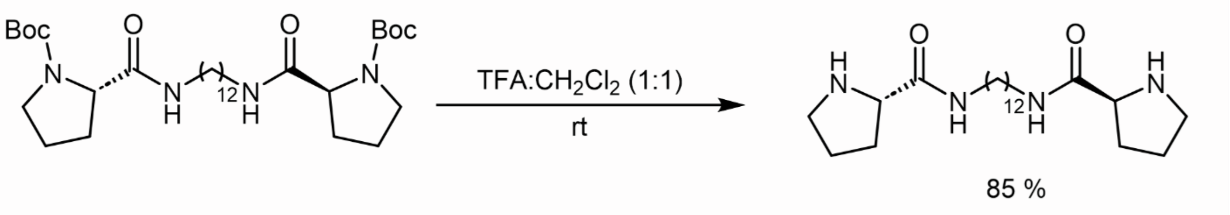

Using **SI-3** (0.390 g, 0.656 mmol) furnished **2Pro-C12:0** (0.220 g, 85%) as a white solid after workup.

R_f = 0.21 (9:1 EtOAc/MeOH); ^{1}H NMR (500 MHz, $CDCl_3$) δ 7.62–7.52 (m, 2H), 3.71 (dd, J = 9.4, 5.3 Hz, 2H), 3.24–3.18 (m, 4H), 3.03–2.97 (m, 2H), 2.91–2.85 (m, 2H), 2.16–2.07 (m, 2H), 1.96–1.86 (m, 4H), 1.76–1.64 (m, 4H), 1.53–1.43 (m, 4H), 1.34–1.20 (m, 16H); ^{13}C NMR (125 MHz, $CDCl_3$) δ 175.0, 60.7, 47.3, 38.9, 30.8, 29.7, 29.6, 29.3, 27.0, 26.2; HRMS (ESI) calcd for $C_{22}H_{43}N_4O_2$ $[M + H]^+$ 395.3381, found 395.3383.

Synthesis of 2Pro-C18:1

SI-4

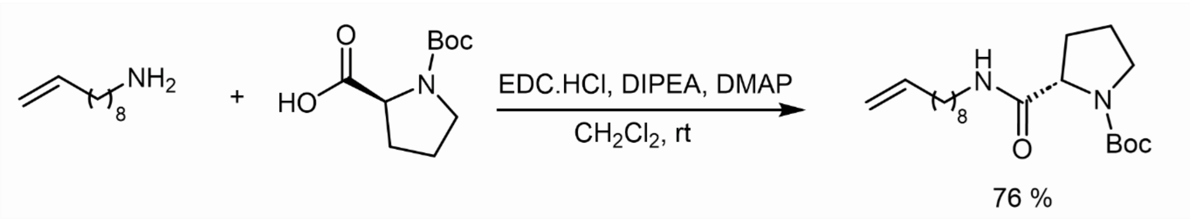

To a solution of amine (3.17 g, 20.4 mmol) Boc-L-proline (4.83 g, 22.5 mmol), DMAP (0.249 g, 2.04 mmol), and DIPEA (4.27 mL, 24.5 mmol) in CH_2Cl_2 (50.0 mL) was added EDC•HCl (4.30 g, 22.5 mmol) at rt. The reaction mixture was stirred at rt for 24 h, after which 1 M HCl (50.0 mL) was added, and the layers separated. The aqueous layer was extracted with CH_2Cl_2 (3 x 30 mL), and the combined organic layers were washed with brine (1 x 50 mL), dried over Na_2SO_4, filtered, and concentrated. Purification by silica gel chromatography (9:1 EtOAc/MeOH) provided amide **SI-4** (1.38 g, 76% yield) as a yellow oil.

R_f = 0.72 (9:1 EtOAc/MeOH); ^{1}H NMR (400 MHz, $CDCl_3$, 333 K) δ 6.54–6.36 (m, 1H), 5.86–5.73 (m, 1H), 5.01–4.89 (m, 2H), 4.26–4.19 (m, 1H), 3.43–3.36 (m, 2H), 3.26–3.18 (m, 2H), 2.07–1.99 (m, 2H), 1.90–1.82 (m, 2H), 1.52–1.42 (m, 13H), 1.35–1.25 (m, 10H); ^{13}C NMR (100 MHz, $CDCl_3$, 333 K) δ 172.1, 155.5, 139.1, 114.2, 80.4, 60.8, 47.2, 39.5, 33.8, 29.7, 29.4, 29.3, 29.1, 29.0, 28.5, 26.9, 24.3; HRMS (ESI) calcd for $C_{20}H_{37}N_2O_3$ $[M + H]^+$ 353.2799, found 353.2803.

SI-5

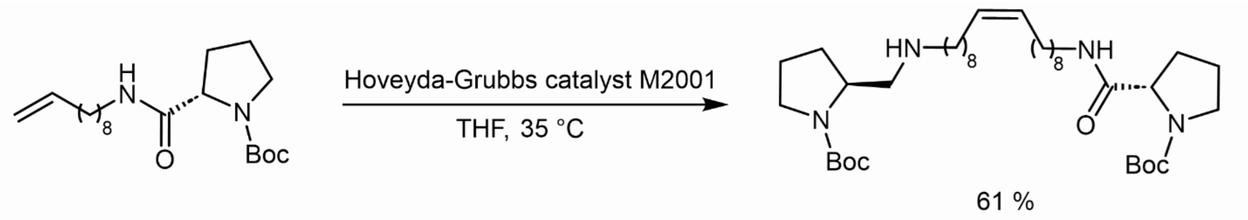

Amide **SI-4** (5.45g, 15.5 mmol) was dissolved in 15.5 mL of dry, degassed THF, and Hoveyda-Grubbs Catalyst® M2001 was added to the reaction mixture, which was stirred at 35 °C for 5 h. The

resulting mixture was then concentrated under reduced pressure and then purified by silica gel chromatography (100% EtOAc) to provide amide **SI-5** (3.17g, 61% yield) as a yellow oil.

R_f = 0.23 (100% EtOAc); ^{1}H NMR (400 MHz, $CDCl_3$, 333 K) δ 6.59–6.31 (m, 1H), 5.38–5.30 (m, 2H), 4.26–4.20 (m, 2H), 3.44–3.38 (m, 4H), 3.27–3.19 (m, 4H), 2.35–2.23 (m, 2H), 2.04–1.98 (m, 6H), 1.90–1.81 (m, 4H), 1.51–1.44 (m, 26 H), 1.37–1.26 (m, 16H); ^{13}C NMR (100 MHz, $CDCl_3$, 333 K) δ 172.1, 155.5, 129.9, 80.4, 60.8, 47.2, 39.5, 29.81, 29.77, 29.5, 29.3, 28.5, 27.3, 27.0, 24.3; HRMS (ESI) calcd for $C_{38}H_{68}N_4NaO_6$ $[M + Na]^+$ 699.5031, found 699.5035.

2Pro-C18:1

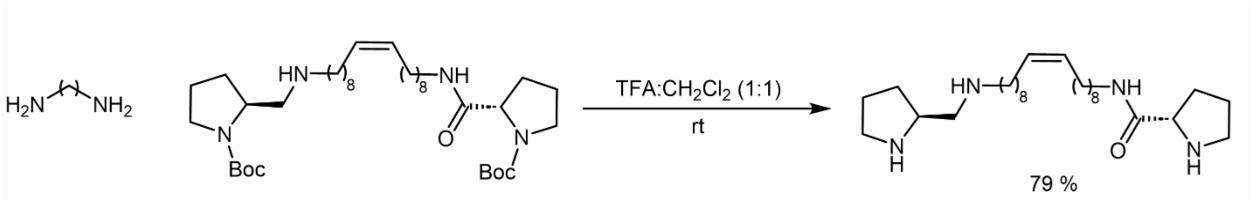

Amide **SI-5** (3.17g, 5.33 mmol) was dissolved in a 1:1 mixture of CH_2Cl_2:TFA (15 mL:15 mL) and stirred at rt for 24 h, after which the resulting mixture was concentrated under reduced pressure. The crude mixture was then dissolved in CH_2Cl_2 (30 mL) and washed with 1 M $NaOH_{(aq)}$ (1 x 30 mL). The aqueous layer was extracted with CH_2Cl_2 (3 x 20 mL), and the combined organic extracts were washed with H_2O (1 x 35 mL), brine (1 x 35 mL), and then dried over Na_2SO_4. Filtration and evaporation of the solvent yielded amide **2Pro-C18:1** (1.65 g, 79%) as a pure yellow oil.

R_f = 0.43 (9:1 EtOAc/MeOH); ^{1}H NMR (500 MHz, $CDCl_3$) δ 7.61–7.52 (m, 1H), 5.33–5.29 (m, 2H), 3.69 (d, J = 9.3, 5.3 Hz, 2H), 3.21–3.15 (m, 4H), 3.01–2.95 (m, 2H), 2.89–2.83 (m, 2H), 2.14–2.05 (m, 4H), 2.01–1.85 (m, 6H), 1.73–1.63 (m, 4H), 1.50–1.41 (m, 4H), 1.34–1.21 (m, 20H); ^{13}C NMR (100 MHz, $CDCl_3$) δ 174.9, 129.8, 60.6, 47.3, 38.9, 30.8, 29.71, 29.68, 29.4, 29.3, 29.2, 27.2, 26.9, 26.2; HRMS (ESI) calcd for $C_{28}H_{53}N_4O_2$ $[M + H]^+$ 477.4163, found 477.4163.

Synthesis of 1Pro-C18:1

SI-6

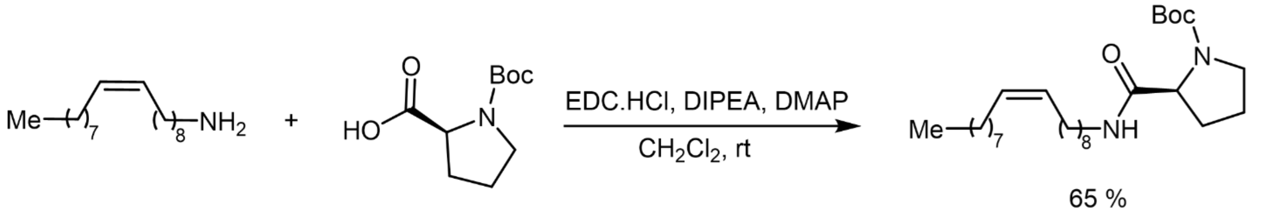

To a solution of oleylamine (2.50 g, 9.35 mmol), Boc-L-proline (2.21 g, 10.3 mmol), DMAP (0.114 g, 0.935 mmol), and DIPEA (1.79 mL, 10.3 mmol) in CH_2Cl_2 (25.0 mL) was added EDC•HCl (1.97 g, 10.3 mmol at rt. The reaction mixture was stirred at rt for 24 h, after which 1 M HCl (25.0 mL) was added, and the layers separated. The aqueous layer was extracted with CH_2Cl_2 (3 x 20 mL), and the combined organic layers were washed with brine (1 x 50 mL), dried over Na_2SO_4, filtered, and concentrated. Purification by silica gel chromatography (1:1 hexanes/EtOAc) provided amide **SI-6** (2.80 g, 65% yield) as a yellow oil.

1Pro-C18:1

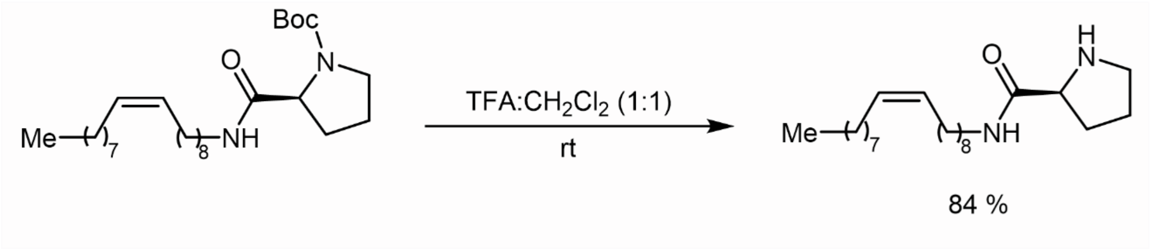

Amide **SI-6** (2.80 g, 6.02 mmol) was dissolved in a 1:1 mixture of CH_2Cl_2:TFA (10 mL:10 mL) and stirred at rt for 24 h, after which the resulting mixture was concentrated under reduced pressure. The crude mixture was then dissolved in CH_2Cl_2 (15 mL) and washed with 1 M $NaOH_{(aq)}$ (1 x 30 mL). The aqueous layer was extracted with CH_2Cl_2 (3 x 15 mL), and the combined organic extracts were washed with H_2O (1 x 30mL), brine (1 x 30 mL), and then dried over Na_2SO_4. Filtration and evaporation of the solvent yielded amide **1Pro-C18:1** (1.85 g, 84%) as a pure yellow oil:

R_f = 0.62 (9:1 EtOAc/MeOH); ^1H NMR (500 MHz, $CDCl_3$) δ 7.67–7.55 (m, 1H), 5.38–5.30 (m, 2H), 3.74 (dd, *J* = 9.2, 5.5 Hz, 1H), 3.23–3.16 (m, 2H), 3.04–2.98 (m, 1H), 2.92–2.86 (m, 1H), 2.69–2.59

(m, 1H), 2.16–2.08 (m, 1H), 2.02–1.85 (m, 4H), 1.74–1.65 (m, 2H), 1.51–1.43 (m, 2H), 1.35–1.19 (m, 22H); ^{13}C NMR (125 MHz, $CDCl_3$) δ 174.6, 130.0, 129.8, 60.6, 47.2, 39.0, 32.6, 31.93, 31.91, 30.8, 29.8, 29.75, 29.70, 29.66, 29.5, 29.4, 29.1, 27.22, 27.21, 26.9, 26.1, 22.7; HRMS (ESI) calcd for $C_{23}H_{45}N_2O$ $[M + H]^+$ 365.3526, found 365.3526.
